# Synthesis of vacant graphitic carbon nitride in argon atmosphere and its utilization for photocatalytic hydrogen generation

**DOI:** 10.1038/s41598-022-17940-3

**Published:** 2022-08-10

**Authors:** Petr Praus, Lenka Řeháčková, Jakub Čížek, Aneta Smýkalová, Martin Koštejn, Jiří Pavlovský, Miroslava Filip Edelmannová, Kamila Kočí

**Affiliations:** 1 Department of Chemistry and Physico-Chemical Processes, VŠB-Technical University of Ostrava, 17. listopadu 15, 708 00 Ostrava-Poruba, Czech Republic; 2 Institute of Environmental Technology, CEET, VŠB-Technical University of Ostrava, 17. listopadu 15, 708 00 Ostrava-Poruba, Czech Republic; 3 Department of Low-Temperature Physics, Faculty of Mathematics and Physics, Charles University, V Holešovičkách 2, Prague 8, Czech Republic; 4 Institute of Chemical Process Fundamentals, Czech Academy of Science, Rozvojová 1, 165 02 Prague, Czech Republic

**Keywords:** Nanoscale materials, Two-dimensional materials

## Abstract

Graphitic carbon nitride (C_3_N_4_) was synthesised from melamine at 550 °C for 4 h in the argon atmosphere and then was reheated for 1–3 h at 500 °C in argon. Two band gaps of 2.04 eV and 2.47 eV were observed in all the synthetized materials. Based on the results of elemental and photoluminescence analyses, the lower band gap was found to be caused by the formation of vacancies. Specific surface areas of the synthetized materials were 15–18 m^2^g^−1^ indicating that no thermal exfoliation occurred. The photocatalytic activity of these materials was tested for hydrogen generation. The best photocatalyst showed 3 times higher performance (1547 μmol/g) than bulk C_3_N_4_ synthetized in the air (547 μmol/g). This higher activity was explained by the presence of carbon (V_C_) and nitrogen (V_N_) vacancies grouped in their big complexes 2V_C_ + 2V_N_ (observed by positron annihilation spectroscopy). The effect of an inert gas on the synthesis of C_3_N_4_ was demonstrated using Graham´s law of ammonia diffusion. This study showed that the synthesis of C_3_N_4_ from nitrogen-rich precursors in the argon atmosphere led to the formation of vacancy complexes beneficial for hydrogen generation, which was not referred so far.

## Introduction

Graphitic carbon nitride is a metal-free semiconductor, which has been intensively studied since 1989 when Liu and Cohen^1^ theoretically predicted a new class of hard materials. Well-known properties, such as the narrow band gap of 2.7 eV^2,3^ and the favourable positions of valence and conduction bands^4^, make this material interesting for various applications in photocatalysis^5,6^, solar cells fabrication^7^, imaging, biotherapy, and sensing of some compounds^8–11^. On the other hand, there are also drawbacks of this material, such as low specific surface area and fast recombination of photoinduced electrons and holes, which can be overcome with its exfoliation^12–14^, doping^15–17^ and the formation of heterostructure composite materials^10,18^. The properties and synthetic procedures have been reviewed in many comprehensive papers, for example^19–27^.

C_3_N_4_ has been mostly synthetized by heating of nitrogen-rich precursors in air^28^ and other atmospheres but only a few papers studied the influence of the atmosphere on its structural, textural, optical and photocatalytic properties. For example, there are papers dealing with the synthesis in reducing hydrogen^29–31^ or inert nitrogen^32–35^ and argon atmospheres^36,37^. The synthesis of C_3_N_4_ in the hydrogen or inert atmosphere was employed in defect engineering^28,29,32,34,37^ mostly for the photocatalytic generation of hydrogen. Comprehensive reviews of the defect engineering have been published recently in the literature as well^38–41^.

Recently, we studied the synthesis of C_3_N_4_ from melamine under air and nitrogen^33^. The aim of this work was to continue with the C_3_N_4_ synthesis in argon (CN-Ar) and to compare obtained results with the previous ones. Effect of the argon atmosphere on the physico-chemical properties of the CN-Ar materials was studied by common characterization techniques and positron annihilation spectroscopy (PAS). Their photocatalytic activity in terms of hydrogen generation was studied as well. The formation of complex vacancies was found and a simple model for the diffusion of NH_3_ released during the synthesis in an inert gas was derived.

## Materials and methods

### Chemicals

All chemicals used were of analytical-reagent grade. Melamine was obtained from Sigma-Aldrich (Darmstadt, Germany). Distilled water was used for the preparation of all solutions and experiments.

### Synthesis of reference C_3_N_4_

Reference C_3_N_4_ used for comparison (labelled as CN) was synthetized by heating of melamine in an ambient air atmosphere in a ceramic crucible with a lid (diameter 5 cm, 30 mL), starting from the ambient temperature with the heating rate of 3 °C min^−1^ up to 550 °C. The total time of synthesis was adjusted to 4 h. The crucible was removed from the muffle furnace and allowed to cool down to the ambient temperature. The CN material was collected and then ground in an agate mortar to a fine powder.

For comparison, CN was further heated in air at 500 °C for 1–3 h with the heating rate of 10 °C min^−1^. The ceramic plate with the product was cooled down to the ambient temperature out of the furnace. Then, the product was pulled out, cooled in a desiccator to the ambient temperature, and ground in the agate mortar to a fine powder. These reheated materials were labelled as CN-1, CN-2, and CN-3.

### Synthesis of CN-Ar materials

Bulk C_3_N_4_ labelled as CN-Ar0 was synthesized by heating of melamine in a tube furnace CLASIC from the ambient temperature to 550 °C at the heating rate of 3 °C min^−1^. Melamine was placed in a ceramic crucible, closed with a lid, and placed in the furnace, which was hermetically sealed, evacuated to the pressure of 0.1 Pa, and then purged with argon of high purity (> 99.9999%). The last two steps were repeated. The temperature was monitored with a Pt-13% Rh/Pt thermocouple located close to the sample. The total synthesis time, including 1 h of dwell at 550 °C, was 4 h, with the argon continuous flow rate of 2 L min^−1^. After that, the product was pulled out, cooled in a desiccator to the ambient temperature, and ground in the agate mortar to a fine powder.

In order to perform further heating of CN-Ar0 we placed it in a ceramic combustion boat and heated it from the ambient temperature to 500 °C at the heating rate of 10 °C min^−1^. The temperature of 500 °C was then kept for 1 (CN-Ar1), 2 (CN-Ar2), and 3 (CN-Ar3) hours. The furnace was flushed with argon having the flow rate of 2 L min^−1^. Then, the reheated CN-Ar materials were placed in a desiccator to cool down to the ambient temperature. Finally, the materials were ground to fine powders.

### UV–vis diffuse reflectance spectroscopy

UV–Vis diffuse reflectance spectra (DRS) were recorded with a spectrophotometer Shimadzu UV-2600 (IRS-2600Plus, Japan) in the range of 220–1000 nm. The reflectance spectrum was transformed to the Kubelka–Munk function F(R) as follows:

1 $$F\left( R \right) = \frac{{\left( {1 - {\text{R}}} \right)^{2} }}{2R}$$

where *R* is the diffuse reflectance from a semi-infinite layer. The values of band gap energies (*E*_*g*_) were determined according to well-known Tauc procedure^42^ as follows:

2 $$\varepsilon h\nu = C\left( {h\nu - E_{g} } \right)^{p}$$

where *ε* is the molar extinction coefficient, *hν* is the energy of incident photons, *C* is a constant and *p* is a power depending on the type of electron transition. The power *p* = 2 and *p* = ½ are for direct and indirect semiconductors, respectively. In this work *p* = ½^43^.

### Photoluminescence spectroscopy

Photoluminescence (PL) and excitation spectra were recorded by a FLSP920 Series spectrometer (Edinburgh Instruments, UK) using an Xe900 arc non-ozone lamp 450 W (Steady State Lamp) and an R928P PMT detector. The PL spectra were measured in the range from 400 to 600 nm. The individual spectra were measured with the excitation wavelength of 368 nm and with the excitation slit of 0.4 nm, the emission slit of 0.4 nm and with the dwell time of 0.5 s.

### Elemental analysis

The elemental composition of the synthetized materials was determined by a Flash 2000 Elemental analyser (Thermo Fisher Scientific, Waltham, MA, USA). The contents of carbon, nitrogen and hydrogen were measured, and the content of oxygen was calculated as a difference of 100%.

### X-ray diffraction analysis

The X-ray diffraction (XRD) analysis was carried out by means of a Rigaku SmartLab diffractometer (Rigaku, Tokyo, Japan) with a detector D/teX Ultra 250. A source of X-ray irradiation was a Co tube (CoKα, λ_1_ = 0.178892 nm, λ_2_ = 0.179278 nm) operated at 40 kV and 40 mA. XRD patterns were recorded between 5° and 90° of 2θ with the step size of 0.01° and the speed of 0.5 deg min^-1^. The crystallite size *L* was calculated using Scherrer´s equation for broadening *B *(2*θ*) (in radians) at a half maximum intensity (FWHM) of a diffraction band as

3 $$B\left( {2\theta } \right) = \frac{K\lambda }{{L\cos \theta }}$$

where *λ* is the wavelength of X-rays, *θ* is Bragg´s angle and *K* is the constant equal to 0.94 for cube or 0.89 for spherical crystallites. In this work *K* = 0.90.

### Fourier transform infrared spectroscopy

Fourier transform infrared (FTIR) spectroscopy was performed using a Nicolet iS50 device (Thermo Scientific, Waltham, MA, USA). The samples were prepared by the KBr pellet technique. A small amount of sample was mixed and homogenised with KBr (approximately 200 mg) and pressed at the pressure of 20 MPa to obtain a transparent tablet. FTIR spectra were collected in the range of 500–4000 cm^−1^ with the resolution of 2 cm^−1^. Each spectrum consisted of at least 64 scans lasting 1 s.

### X-Ray photoelectron spectroscopy

Superficial elemental analysis was performed by means of an X-ray photoelectron spectrometer (XPS) ESCA 3400 (Kratos Analytical Ltd, UK) with the base pressure in the analysis chamber of 5.0 * 10^−7^ Pa. Powdered materials were placed on top of a conductive carbon tape. Electrons were excited with Mg Kα radiation (hν = 1253.6 eV) generated at 12 kV and 10 mA. For all spectra, the Shirley background was subtracted. Peaks ascribed to sp^2^ hybridized nitrogen (C=N–C) were set to 398.8 eV as a charge correction.

### Physisorption of nitrogen

Specific surface area (SSA) was determined by physisorption of nitrogen at − 196 °C. The experiments were performed using a device SORPTOMATIC 1990 series (Thermo Scientific, Waltham, MA, USA). The adsorption–desorption isotherms were evaluated by means of the Brunauer, Emmett, and Teller (BET) method. The pore size distribution was calculated according to the Barrett, Joyner, and Halenda (BJH) model.

### Scanning electron microscopy

Microscopic analysis was performed with a scanning electron microscope Tescan Vega (Tescan Orsay Holding, Brno, Czech Republic) with a tungsten cathode and an energy-dispersive X-ray spectrometer (EDAX, Ametex, PA, USA). SEM micrographs were obtained using a mix of the signals of secondary electrons (SE) and backscattered electrons (BSE) mode to get the benefit of both techniques (SE + BSE).

### Photoelectrochemical measurements

Photoelectrochemical measurements were conducted using a photoelectric spectrometer equipped with a 150 W Xe lamp and coupled with a potentiostat (Instytut Fotonowy, Poland). Photocurrent responses were recorded using a classic three-electrode setup. Ag/AgCl and platinum wires were used as reference and counter electrodes, respectively.

The working electrode was prepared as follows: 20 mg of powdered material was suspended in 150 µL drops of ethanol and ultrasonicated for 15 min. Afterwards, 45 µL of the suspension was deposited onto an indium-tin oxide (ITO) foil and a uniform layer was created using a film applicator (Elcomoter 3570). The foil with the layer was dried at 80 °C resulting in an adsorbed material on an ITO surface creating conductive connection. The 0.1 mol L^−1^ KNO_3_ solution was used as an electrolyte. Photocurrent spectra were recorded within the range of 240–450 nm (with the step of 10 nm) in the presence of the external potential range of 0.2–1.0 V (step 0.1 V).

### Mott-Schottky measurements

Mott-Schottky measurements were performed using a Metrohm Autolab PGSTAT302 (Herisau, Switzerland) potentiostat. A glassy carbon electrode (GC), Ag/AgCl (3 mol L^−1^ KCl) electrode and a Pt sheet served as working, reference and counter electrodes, respectively. All electrodes were purchased from Metrohm. The Mott-Schottky measurements were performed twice with an AC signal having an amplitude of 10 mV and a frequency of 300 Hz. The validity of single-frequency Mott-Schottky measurement was checked by a more rigorous approach involving the determination of surface capacitances from a series of full electrochemical impedance spectra recorded at different potentials (for details refer to Supplementary material).

A thin layer of the materials was prepared on the GCE surface as follows. The powdered CN and CN-Ar materials, each in the amount of 10 mg, were added into 5 mL of deionized water, and then the mixtures were subjected to 30-min sonication in an ultrasonic bath. Then, 30 μL of the dispersion was dropped on the GC surface and dried at 85 °C for 3 h. The samples were measured in the 0.1 mol L^−1^ KCl aqueous solution, which was purged by nitrogen for 30 min before the experiment.

### Photocatalytic experiments

**Figure 1:**
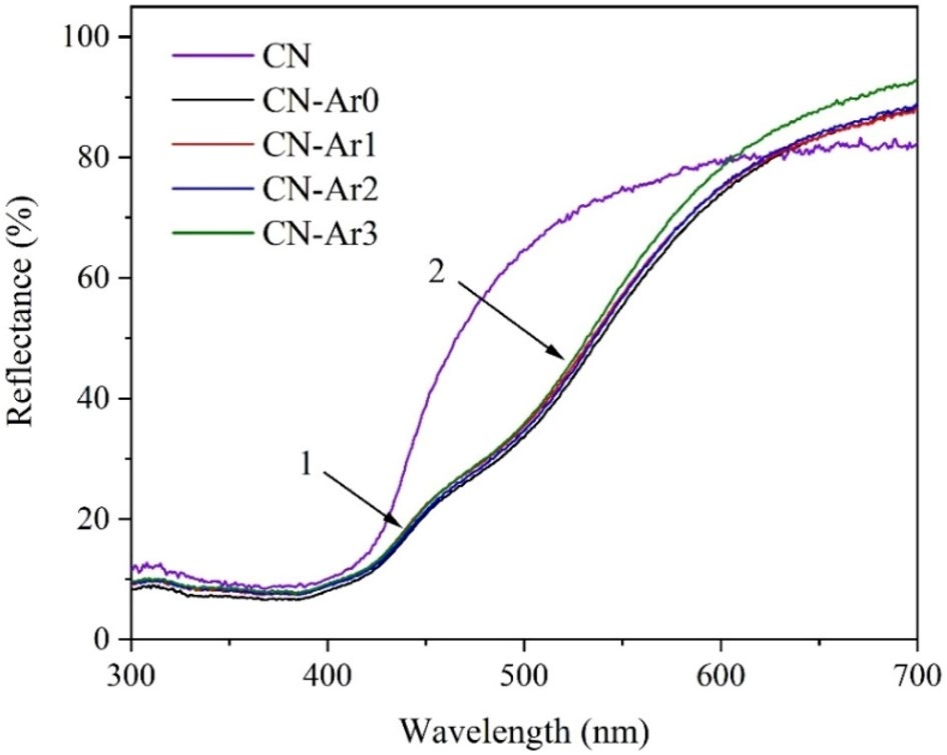
UV–Vis reflectance spectra of CN and CN-Ar materials.

The photocatalytic activity of the CN and CN-Ar materials was investigated in terms of hydrogen generation. The photocatalytic experiments were performed in a stirred batch photoreactor (stainless steel, volume 348 ml, Fig. 1S in Supplementary materials). The reaction mixture contained 100 mL of 50% methanol with a photocatalyst (0.1 g) and was saturated with helium to purge air and to saturate the solution. An 8 W Hg lamp (254 nm; Ultra-Violet Products Inc.) was used as an irradiation source and was placed on a quartz glass window on top of the photoreactor in horizontal position (Fig. 1S). The reactor was tightly closed and before the reaction started (switching on the lamp), a gaseous sample was taken (at time 0 h) through a septum with a syringe. All the gaseous samples were analysed by a gas chromatograph (Shimadzu Tracera GC-2010Plus) equipped with a barrier discharge ionization detector (BID). The reaction mixture was irradiated at certain time intervals (0–4 h) and samples were taken at 1, 2, 3 and 4 h for the GC analysis. Three reaction products were determined: hydrogen, methane, and carbon monoxide.

### Positron annihilation spectroscopy

A ^22^Na positron source with the activity of ≈ 1 MBq deposited on a 2 µm thick Mylar foil was used in PAS measurements. The diameter of the positron source spot was 2 mm. The positron source was placed in the centre of a small cylindrical chamber with the diameter of 10 mm and height of 5 mm. Subsequently, the chamber was completely filled with the measured powder and closed. Dimensions of the chamber ensured that virtually all positrons were thermalized inside the chamber, and thereby annihilated in the studied powder. A digital positron lifetime spectrometer^44^ with the time resolution of 145 ps was employed for the PAS investigations. At least 10^7^ annihilation events were collected in each positron lifetime spectrum. A source contribution consisting of two components with lifetimes of ≈ 368 ps and ≈ 1.5 ns and corresponding intensities of ≈ 8% and ≈ 1% was always subtracted from the spectra. Decomposition of positron lifetime spectra into exponential components was performed using a dedicated code PLRF^45^.

*Ab-initio* theoretical calculations of positron lifetimes were employed in order to identify defects in the CN and CN-Ar materials. Positron lifetimes were calculated using density functional theory within a so-called standard scheme^46^. In this approximation, the positron density is assumed to be vanishingly small everywhere and not affecting the bulk electron structure. At first, electron density *n*_-_(***r***) in the material was being solved without a positron. Subsequently, the effective potential for positron was constructed by the superposition of the Coulomb potential produced by the charge distribution of electrons and nuclei and the electron–positron correlation potential^47^. The ground state positron wave function *Ψ*^+^(***r***) was calculated by solving a single-particle Schrödinger equation for a positron in the effective potential. The positron lifetime was determined using an overlap of the electron and positron densities and positron density $n_{ + } \left( {\varvec{r}} \right) = \left| {\psi^{ + } \left( {\varvec{r}} \right)} \right|^{2}$ through the expression

4 $$\tau = \left[ {\pi r_{e}^{2} c\smallint n_{ + } \left( {\varvec{r}} \right) n_{ - } \left( {\varvec{r}} \right)\gamma \left[ {n_{ - } ,\nabla n_{ - } } \right]d{\varvec{r}}} \right]^{ - 1} ,$$

where *r*_*e*_ is the classic electron radius, *c* is the speed of light, and $\gamma$ denotes the electron enhancement factor describing the pileup of electrons at the positron site. The electron-positrons correlation, i.e. the correlation potential and the enhancement factor $\gamma$, were treated within the generalized gradient approximation (GGA) using the scheme developed by Barbiellini et al.^48,49^.

C_3_N_4_ tri-s-triazine (heptazine) ring-based layered structure was considered in the calculations^26^. Heptazine rings are cross-linked by triangular nitrogen atoms forming a two-dimensional honeycomb lattice. The interlayer distance between the heptazine layers is 3.19 Å^50^ and the lattice parameter of 1 × 1 unit cell is 7.14 Å^51^. *Ab-initio* calculations were performed using 3594 atom-based supercells (consisting of 1536 C and 2048 N ions). Defects were modelled by removing the corresponding number of C or N atoms from the supercell. Convergence tests with respect to the supercell size revealed that the calculated positron lifetimes converged within ± 1 ps.

## Results and discussion

### UV–vis absorption study of CN-Ar materials

**Figure 2:**
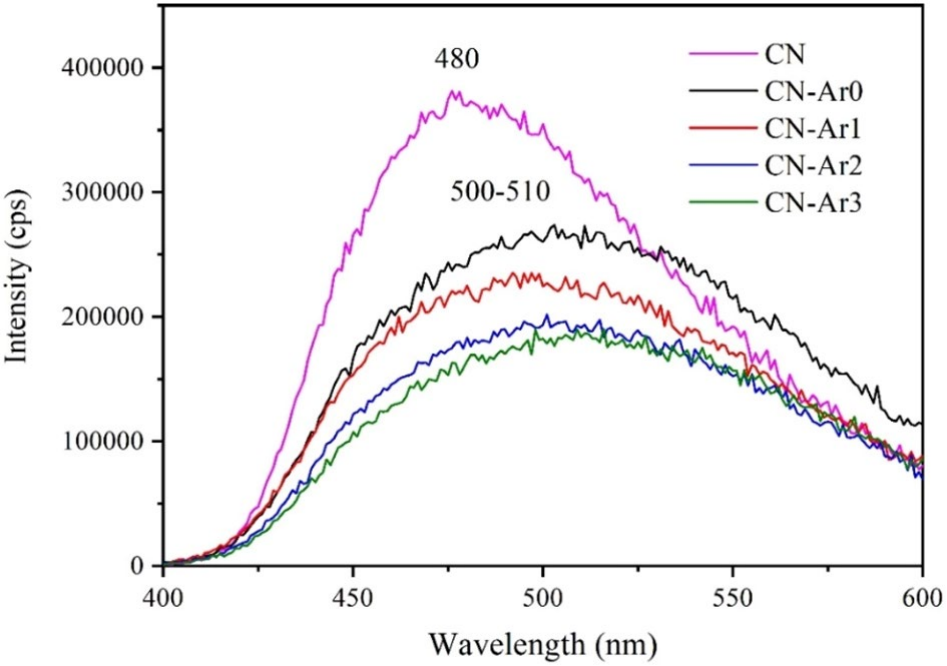
PL spectra of CN and CN-Ar materials.

**Figure 3:**
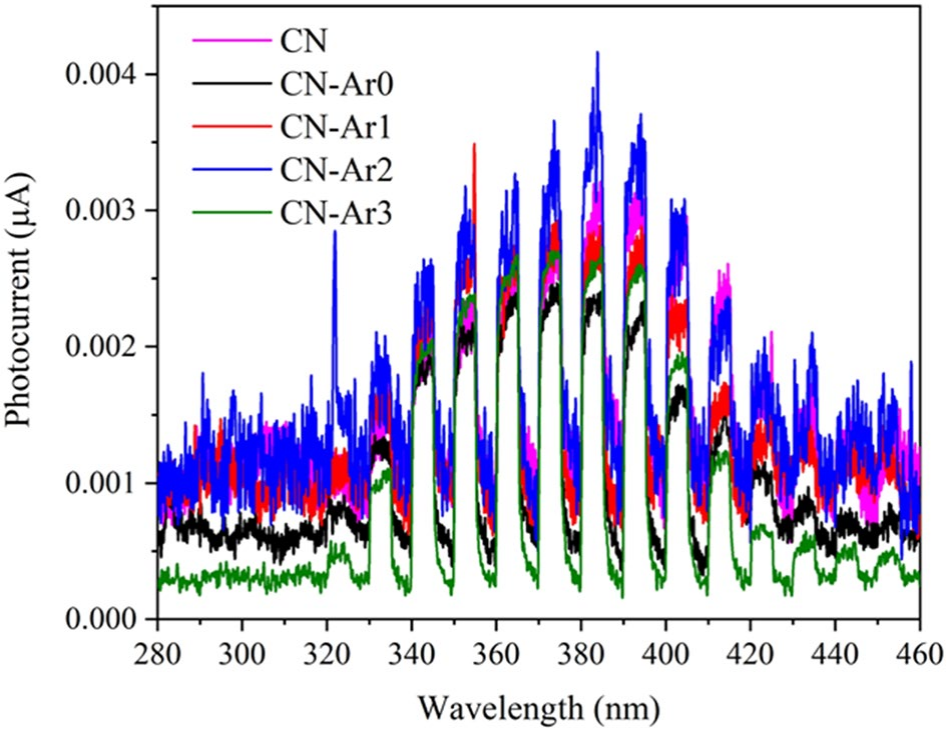
Photocurrents of CN and CN-Ar materials recorded at 1 V versus Ag/AgCl in deoxygenated 0.1 mol L^−1^ KNO_3_.

The optical properties of the CN-Ar materials were studied by UV–Vis DRS, see Fig. 1. The real colours of these materials are displayed in Fig. 2S (Supplementary materials). Two absorption edges labelled 1 and 2 are visible. The corresponding band gap energies were determined according to common Tauc plots displayed in Fig. 3S and were summarized in Table 1.

**Table 1:** Band gap energies and SSA of CN and CN-Ar materials.

Material	E_g_1 (eV)	E_g_2 (eV)	SSA (m^2^ g^−1^)
CN	–	2.65	12
CN-Ar0	2.03	2.46	15
CN-Ar1	2.03	2.48	17
CN-Ar2	2.04	2.46	18
CN-Ar3	2.05	2.47	18

By comparing these band gaps with the SSA changing from 15 to 18 m^2^ g^−1^ (Table 1), it is indicated that no exfoliation occurred during the heating of CN-Ar0 for 1–3 h. If CN-Ar0 were exfoliated, the band gaps would have increased due to a quantum size effect, but it was not observed. The spectrum of the CN material used for comparison demonstrates only one absorption edge corresponding to the band gap of 2.65 eV. The new band gaps of 2.02–2.09 eV were also observed by several authors^29,33^ due to the presence of structure defects. The light absorption extension in UV–Vis DRS spectra was also observed by Lv et al.^28^.

**Figure 4:**
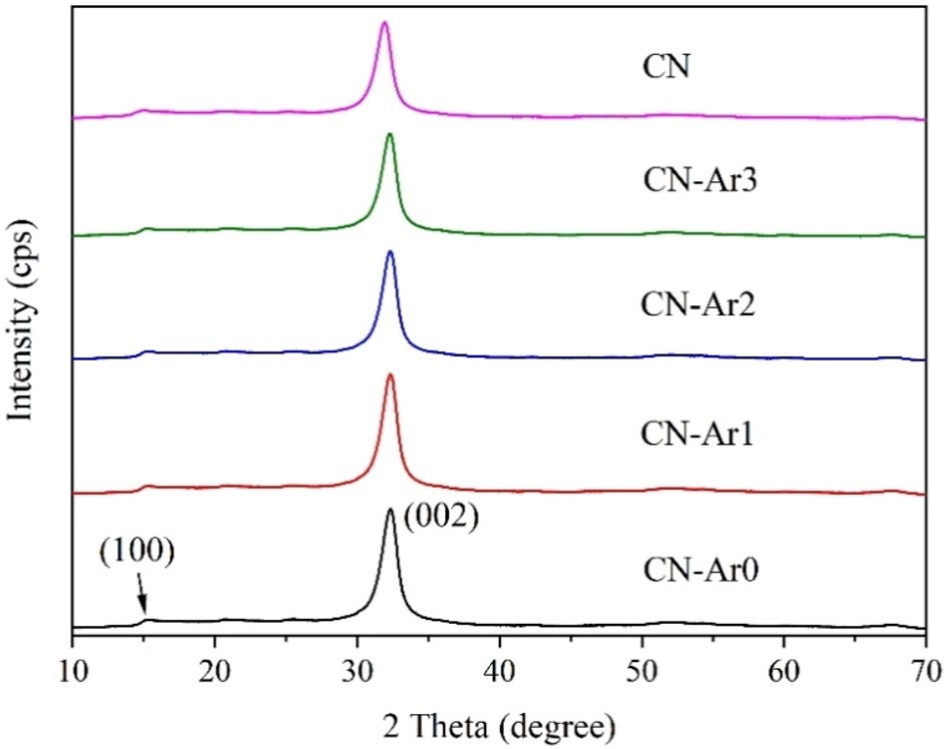
XRD patterns of CN and CN-Ar materials.

The second band gaps of the CN-Ar materials were very similar, that is, from 2.46 to 2.48 eV. On the contrary, the band gaps of the CN materials further heated in air increased from 2.72 to 2.77 eV due to its exfoliation. It was also documented by increasing their SSA values, see Table 1S and Fig. 4S. The CN-Ar materials were further studied by PL, XRD, FTIR and XPS.

### Photoluminescence study

The photoluminescence spectra of the CN and CN-Ar materials were recorded, see Fig. 2. The broad CN band at around 480 nm (2.58 eV) corresponds to transition of photoinduced electrons from a conduction to valence band. The PL bands of the CN-Ar materials were red-shifted at about 500–510 nm (2.48–2.43 eV) and were broader than the PL band of CN. The PL maxima well agree with the band gap energies mentioned above. The red-shift, the band broadening and the PL intensity decrease can be explained by non-radiative transmission of excited electrons to mid-gap levels of N defects^29,30,33,34,52–54^, from which they radiatively return to the valence bands. The PL effects mentioned above ascribed to the N vacancies were observed for C vacancies^55^ as well. Moreover, another contribution to the band broadening and the red shift is the presence of the second band gap of 2.04 eV as a result of nitrogen defects.

In addition, the PL intensity decreased with the increasing time of heating; the PL intensities of CN-Ar2 and CN-Ar3 were similar. This can be caused by the higher number of defects formed during the heating and, thus, the more non-radiative electron transitions. After 3 h of heating, the number of defects did not increase any more. It can be noted that a nitrogen loss of C_3_N_4_ was observed as a result of its annealing^56^ like in this study and due to electron beam irradiation^57^. The annealing of C_3_N_4_ at 650 °C was also found to lead not only to the creation of N vacancies but also to the formation of new C=C bonds in heptazine units^35^ but it was not observed in this work, see below.

**Figure 5:**
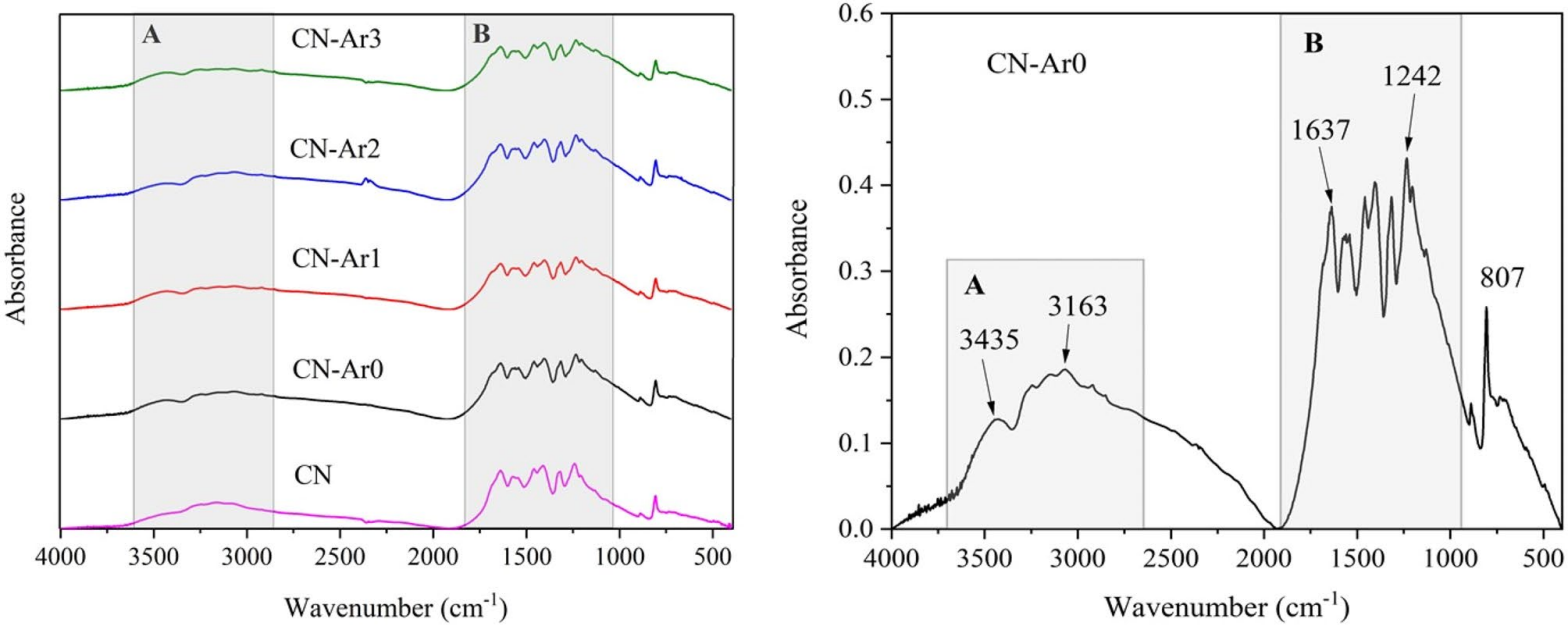
FTIR spectra of CN and CN-Ar materials (left) and spectrum of CN-Ar0 (right).

The decreasing intensity and redshifts of the PL bands mentioned above are opposite to phenomena observed for the CN materials synthesised in air, see Fig. 5S. Their PL bands were blue-shifted, and their intensity increased with the heating time, which we already observed recently^33^. It could be explained by the decreasing number of defects in the CN ones.

### Photoelectrochemical properties

Electric current generated after irradiation of the CN and CN-Ar materials was recorded in order for us to study the photoelectrochemical properties of these materials. The photocurrent generation measurement provides information about the amount of generated charge carriers. The dependence of the generated photocurrent on the wavelength is shown in Fig. 3. The whole measurements were conducted in the range of applied potentials from − 200 to 1000 mV (vs. Ag/AgCl) and in the range of wavelengths for each potential from 240 to 450 nm. The current responses were measured under the maximal applied potential of 1 V to suppress the recombination of photoinduced electrons and holes.

**Figure 6:**
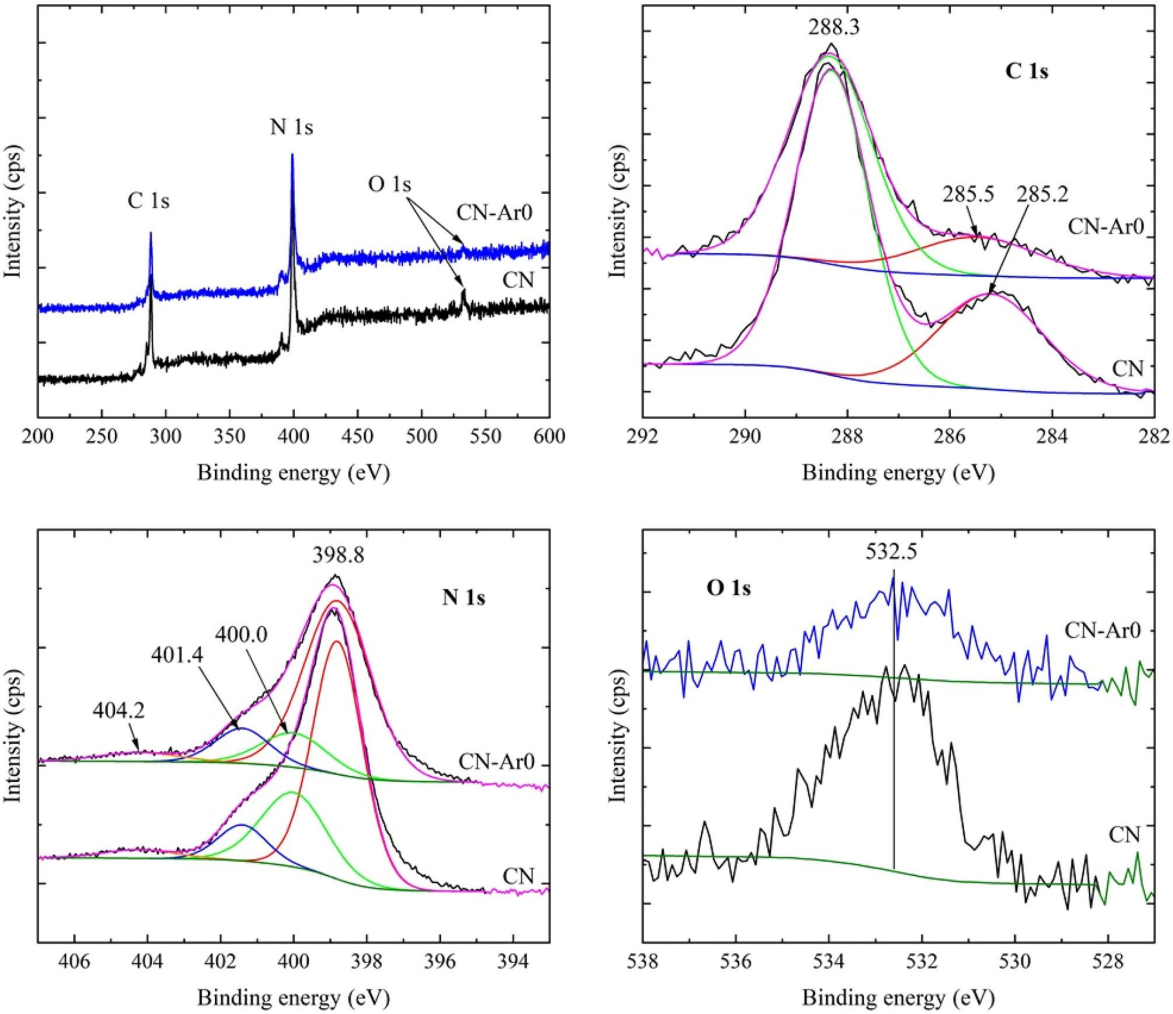
XPS spectra of CN and CN-Ar0 materials.

The maximal photocurrents were measured at about 380 nm within the range of 340–410 nm. The photocurrent records were evaluated based on their signal-to-noise (S/N). In general, when S/N is equal to 3.0 the presence of a significant signal is accepted^58^. The S/N values for 380 nm were calculated at 3.0 for CN, 4.3 for CN-Ar0, 2.9 for CN-Ar1, 3.1 for CN-Ar2 and 8.6 for CN-Ar3; the photocurrents of CN and CN-Ar3 are also shown in Fig. 6S. Both CN and CN-Ar materials were able to generate significant electric current under UVA irradiation.

### X-ray diffraction study

The XRD patterns of the CN and CN-Ar materials were recorded as shown in Fig. 4. The typical diffractions of (002) and (100) planes were observed. The (002) diffractions are attributed to interlayer stacking of C_3_N_4_ planes and the (100) ones are attributed to the in-plane ordering of nitrogen-linked heptazine units^59^.

Some basic characteristics were evaluated from the XRD patterns, see Table 2. The characteristics of all the CN-Ar materials were very similar and no exfoliation was observed in them. Only a little shift of the d(002) spacings between CN and CN-Ar ones was calculated. The crystallite sizes L(002) of about 7 nm were similar for all the materials. One can conclude that no effect of the defects in the CN-Ar structures was observed.

**Table 2:** Basic XRD characteristics of CN and CN-Ar materials.

Material	2 Theta (deg)	FWHM (deg)	L(002) (nm)	d(002) (nm)
CN	31.91	1.30	7.1	0.325
CN-Ar0	32.33	1.32	7.0	0.321
CN-Ar1	32.41	1.29	7.2	0.321
CN-Ar2	32.33	1.28	7.2	0.321
CN-Ar3	32.28	1.28	7.2	0.322

**Figure 7:**
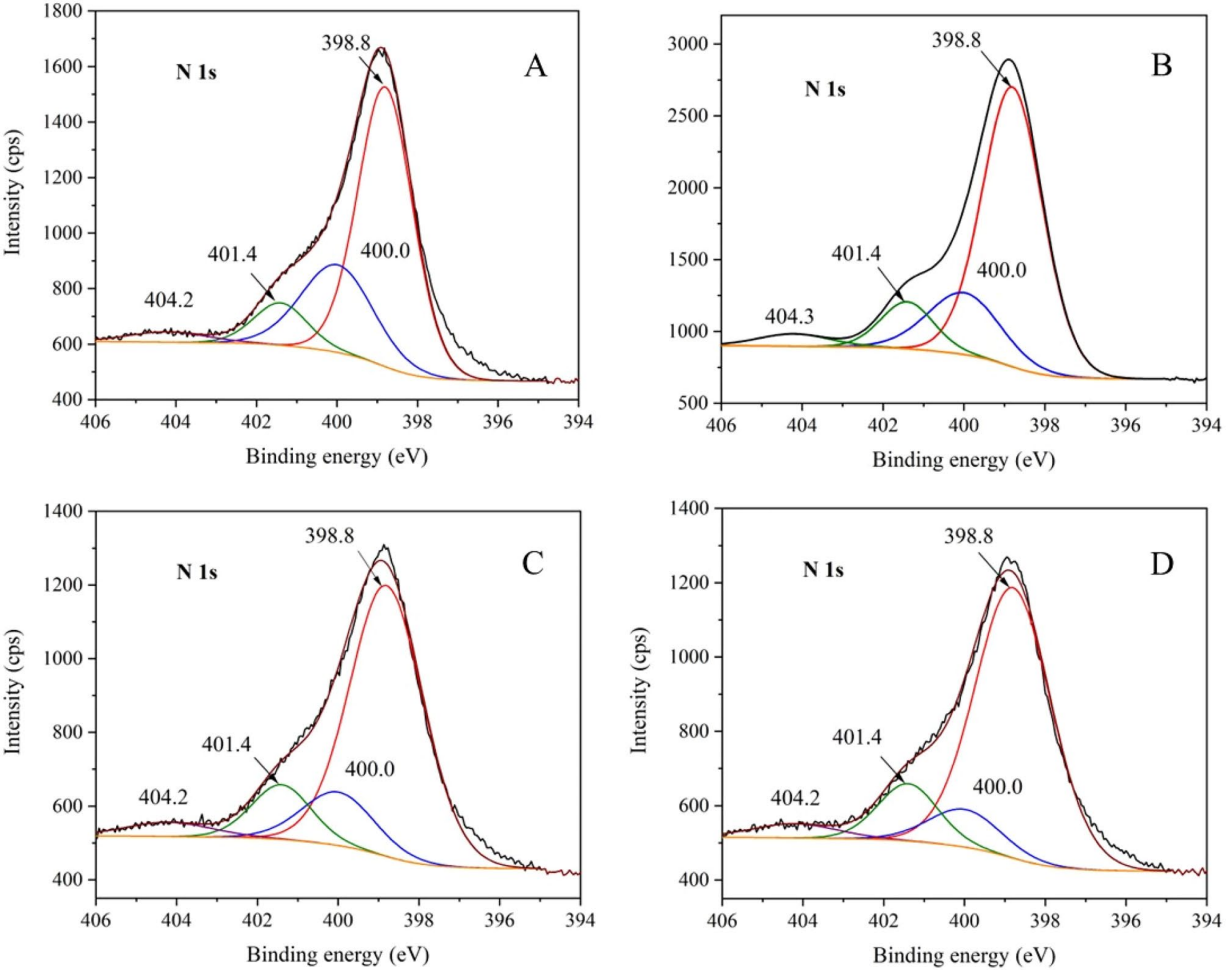
XPS N 1s spectra of C_3_N_4_ synthesised in air and argon. (**A**) CN, (**B**) CN-3, (**C**) CN-Ar0, and (**D**) CN-Ar3.

Similar diffraction patterns (Fig. 7S) were observed for the CN materials synthesised in air, as demonstrated by the basic XRD characteristics in Table 2S. The d(002) spacings were similar to those of the CN-Ar materials (Table 2), and the crystallite sizes L(002) were a little smaller due to the thermal exfoliation providing non-diffracting nanosheets^33^.

### FTIR analysis

The CN and CN-Ar materials were analysed by FTIR to show changes in their structures. Figure 5 displays their FTIR spectra (left) in comparison with that of CN-Ar0 (right). Two band regions A and B can be well distinguished. The bands in the region A are attributed to the stretching vibrations of N–H bonds and those in the region B are explained as the stretching vibrations of C=N and C–N bonds of heterocyclic rings^60^. The band at 807 cm^−1^ identifies the breathing mode of triazine units. A small band at 2350 cm^−1^ of CN-Ar2 was caused by some unidentified impurity. The bands around 3500 cm^−1^ indicate the presence of –OH groups.

In order for us to evaluate the changes of N–H stretching vibrations, the absorbances at 3163 cm^−1^ (primary amines) and 3435 cm^−1^ (secondary amines) were related to those at 1242 cm^−1^ and 1637 cm^−1^ concerning the C–N and C=N stretching vibrations, respectively. Since absorbance is a relative parameter depending on the concentrations of absorbing compounds the ratios of A_3163_/A_1242_ and A_3163_/A_1637_ calculated for the CN and CN-Ar materials can be comparable with each other, see Table 3. Both ratios of CN were lower likely due to the lower content of N–H species in relation to C–N and C=N ones. In other words, the CN-Ar materials had more N–H bonds than the CN one. A possible explanation is in oxidation of –NH_2_ groups during the synthesis in air.

**Table 3:** Ratios of FTIR absorbances of CN and CN-Ar materials.

Material	A_3163_/A_1242_	A_3163_/A_1637_	A_1242_/A_1637_
CN	0.402	0.450	1.12
CN-Ar0	0.429	0.476	1.11
CN-Ar1	0.452	0.494	1.09
CN-Ar2	0.429	0.478	1.11
CN-Ar3	0.449	0.494	1.10

**Figure 8:**
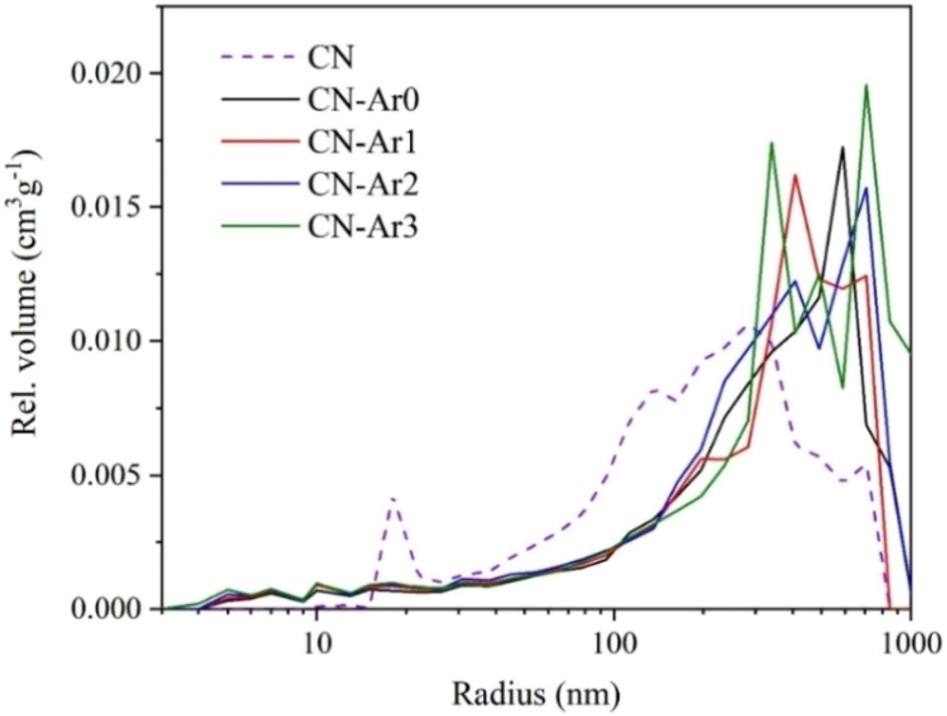
Pore distribution plots of CN and CN-Ar materials.

The FTIR spectra of the CN materials synthesised in the air for 1–3 h were recorded for comparison, see Fig. 8S. The typical band regions A and B and the band at 807 cm^−1^ were also observed. By analysis of absorbance at 3163 cm^−1^, 1242 cm^−1^, and 1637 cm^−1^ one can see that the content of N–H bonds decreased by the reheating in air due to their oxidation, see Table 3S. In addition, the content of C–N bonds in relation to the more stable C=N ones also decreased for the same reason.

Similar ratios of A_1242_/A_1637_ indicate similar composition of heptazine units in the analysed materials. The findings concerning the ratios of A_3435_/A_1242_ and A_3435_/A_1637_ of the N–H vibrations of secondary amines are summarized in Table 4S and they also point out the lower content of N–H species in the CN sample.

### XPS analysis

**Figure 9:**
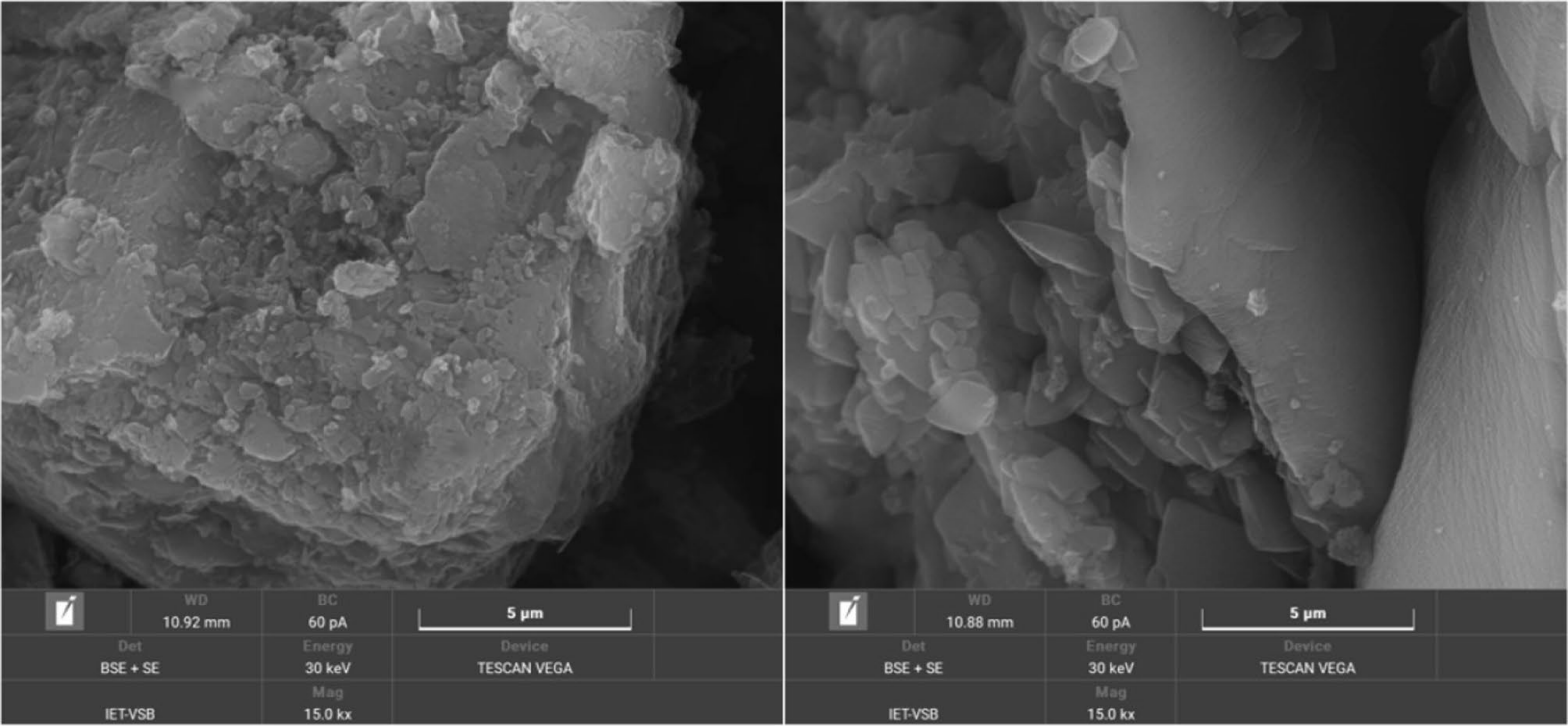
SME (BSE + SE) micrographs of CN (left) and CN-Ar0 (right).

**Figure 10:**
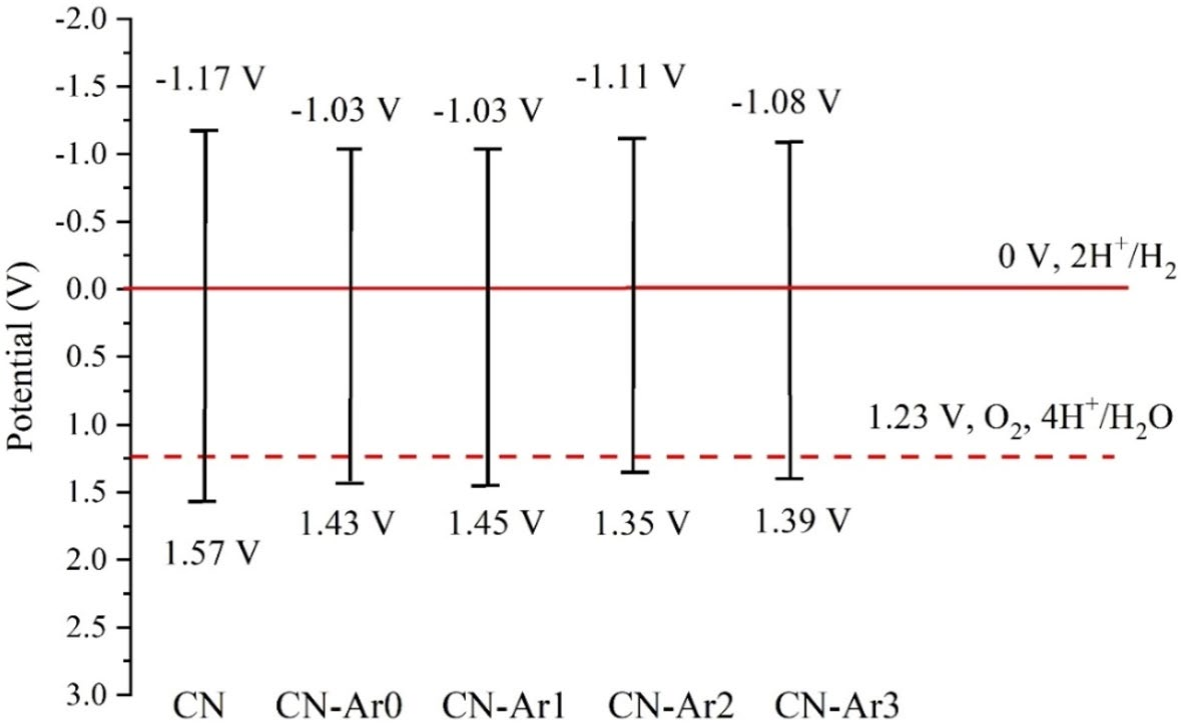
Energy diagram of conduction and valence band potentials of CN and CN-Ar materials at pH = 7.

**Figure 11:**
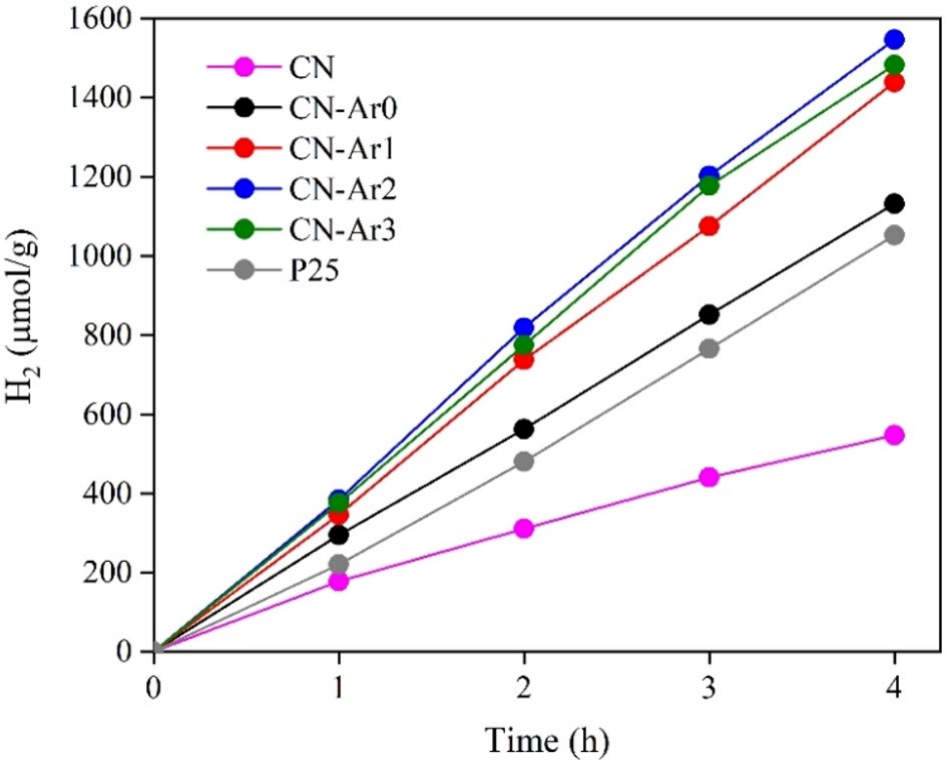
Time dependence of hydrogen yields during the photocatalytic hydrogen generation from water–methanol mixture in the presence of CN, CN-Ar materials and TiO_2_ at irradiation of 254 nm.

The XPS analysis was performed in order for us to see changes in the surface composition and oxidation states of the CN and CN-Ar materials. Whole survey spectra were measured, however, no other element except C, N, and O was found. For deconvolution, detailed C 1s, N 1s and O 1s spectra were used. The spectra of all the CN-Ar materials were similar, therefore, only one of CN-Ar0 is displayed in comparison with CN in Fig. 6. The spectra of the other CN-Ar materials are placed in the Supplementary materials; see Figs. 9S, 10S and 11S. For comparison, the additional N 1s spectra of CN synthesised in air and in argon are demonstrated in Fig. 7, see below.

The deconvolution of the C1s spectra provided no significant differences. These spectra were fitted by two peaks with the positions at 288.3 eV and 285.0–285.5 eV. The peak at 288.3 eV can be ascribed to sp^2^ hybridized carbon (N–C=N). The second peak can be composed of several contributions, such as C–C (284.8 eV), C–O (286–287 eV) or C–N (286 eV). The origin of this peak can be ascribed either to oxidative changes in the CN-Ar materials or/and to adventitious carbon on the surface.

The deconvolution of the N 1s spectra (Fig. 6) showed four peaks positioned at 398.8, 400.0, 401.4 and 404.2 eV. These peaks can be ascribed to sp^2^ hybridized nitrogen (C=N–C) also called two-coordination nitrogen (N_C2_), nitrogen of tertiary amine N-(C)_3_ also called three-coordination nitrogen (N_C3_), the C–N–H bond, and to a π–π* (HOMO–LUMO) transition (shake-up line), respectively. Moreover, the band gap of about 2.04 eV (Table 1) can be explained by the presence of the nitrogen vacancies due to the preferential loss of N_2C_ compared to N_3C_^29,61–63^. The energies for removing N atoms in N_2C_ and N_3C_ are 1.40 eV and 2.39 eV, respectively^29^. The oxygen O1s spectra did not provide data suitable for the deconvolution.

Figure 7 provides the comparison of N 1s spectra of CN, CN-3, CN-Ar0, and CN-Ar3. The different intensities of peaks at 401.4 eV and 400.0 eV concerning N_C3_ and N_C2_ nitrogen atoms, respectively, are remarkable. The decrease of N_C2_ intensities in relation to N_C3_ ones in air and in argon implies the formation of N_C2_ vacancies with the heating time. Moreover, it is also visible that the CN-Ar materials contained a higher portion of N_C2_ than the CN ones synthesised in air. This can be explained by the preferential oxidation of amino groups (N_C3_).

The results of the surface elemental analysis are summarized in Table 4. There are no significant changes in the surface composition of nitrogen and carbon of the CN and CN-Ar materials. Taking into account the experimental error of about 10%, the C/N ratios could be considered to be similar and, therefore, no significant differences in the surface composition of CN-Ar materials were found. However, there are differences in the content of oxygen between the CN and CN-Ar ones. The higher content of oxygen in CN can be explained by surface oxidation during its synthesis in air.

**Table 4:** Elemental surface (XPS) analysis of CN and CN-Ar materials.

Material	C (at %)	N (at %)	O (at %)	C/N	C/N*
CN	47.43	48.93	3.64	0.97	0.67
CN-Ar0	42.67	54.74	2.59	0.78	0.62
CN-Ar1	44.15	53.24	2.61	0.83	0.58
CN-Ar2	41.41	56.59	2.00	0.73	0.51
CN-Ar3	44.16	53.89	1.95	0.82	0.59

*The C/N fragments were calculated using only sp^2^ C at 288.3 eV.

The surface analysis performed by XPS (Table 4) was compared with the bulk elemental analysis, see Table 5. The C/N value of the CN material was lower than the values of the CN-Ar ones (proved by the Dean-Dixon test and a box plot) likely due to the lower content of nitrogen as a result of nitrogen defects formed in the argon atmosphere. This is in consistency with the XPS analysis. The higher content of hydrogen in the CN-Ar materials agrees with the higher portion of > NH and –NH_2_ groups indicated by FTIR.

**Table 5:** Bulk elemental analysis of CN and CN-Ar materials.

Material	C (wt %)	N (wt %)	H (wt %)	O (wt %)	C/N (mol/mol)
CN	34.57	61.57	1.58	2.28	0.655
CN-Ar0	35.10	60.90	1.95	2.05	0.672
CN-Ar1	35.00	60.80	1.91	2.29	0.672
CN-Ar2	35.10	61.10	1.86	1.94	0.670
CN-Ar3	35.20	61.30	1.87	1.63	0.670

It is interesting to see that the content of oxygen is similar in CN and CN-Ar materials, about 2 wt.%. There are questions (i) how oxygen could get in CN-Ar0 if it was synthesised in the argon atmosphere and (ii) why the content of oxygen did not increase when CN-Ar0 was further heated for 1–3 h in argon. A possible answer to the first question is that the incomplete polymerization of melamine could lead to the formation of structural defects, which were attacked with oxygen and water when CN-Ar0 came into contact with air. The second question can be answered by the high thermal stability of the CN-Ar0 structure, which was not changed by the repeated heating at 500 °C for 1–3 h, which is in contrast to the same procedure applied on C_3_N_4_ in air when its exfoliation happed^12,33^. It indicates that the heating alone is not the only reason for exfoliation.

The bulk elemental analysis was also performed on the CN materials synthesised in air, see Table 5S. The content of oxygen increased due to direct oxidation with oxygen. The presence of oxygen attacking C_3_N_4_ and forming its defective structure is natural. The C/N ratios were similar due to oxidation and consequent decarboxylation^33^.

### Texture and morphology study

The CN and CN-Ar materials were analysed in terms of SSA as mentioned above (Table 1) and pore size distribution. It is obvious that the SSA changed very little and the heating time in the argon atmosphere was not important. This is in line with our recent experiments of the C_3_N_4_ synthesis under nitrogen^33^. Unlike the SSA, the pore size distribution plots showed some changes, see Fig. 8. In comparison with CN, the CN-Ar materials had fewer mesopores and more macropores with the radius above 200 nm. The CN mesopores were supposed to be created by erosion of the C_3_N_4_ structure due to its oxidation and consequent decarboxylation in air.

**Figure 12:**
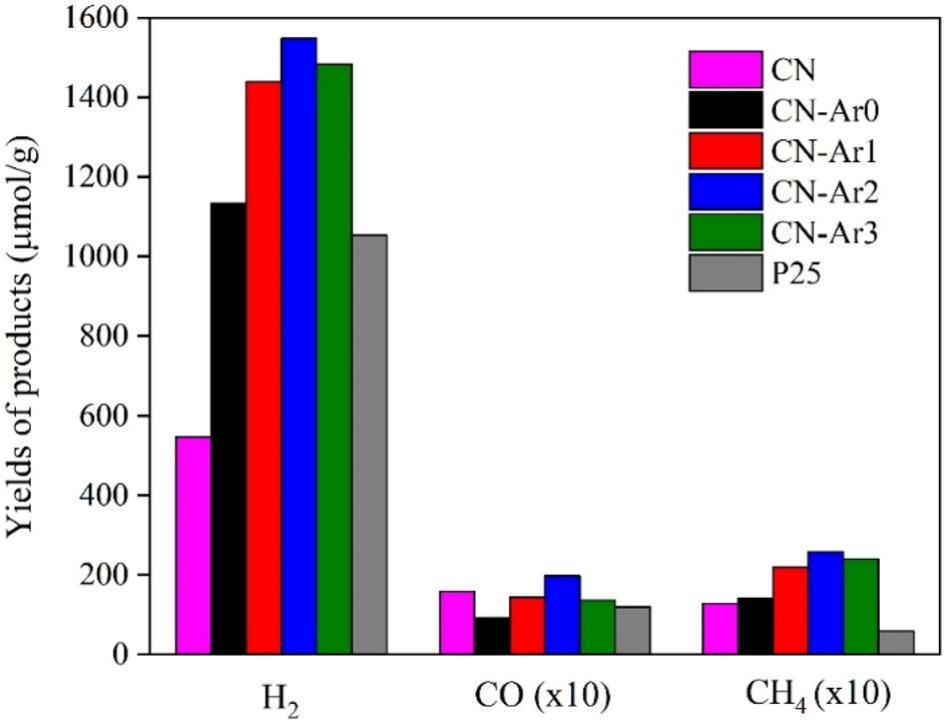
Amount of produced H_2_, CH_4_* and CO* yields after 4 h of irradiation in the presence CN, CN-Ar materials and TiO_2_ (*multiplied by 10).

**Figure 13:**
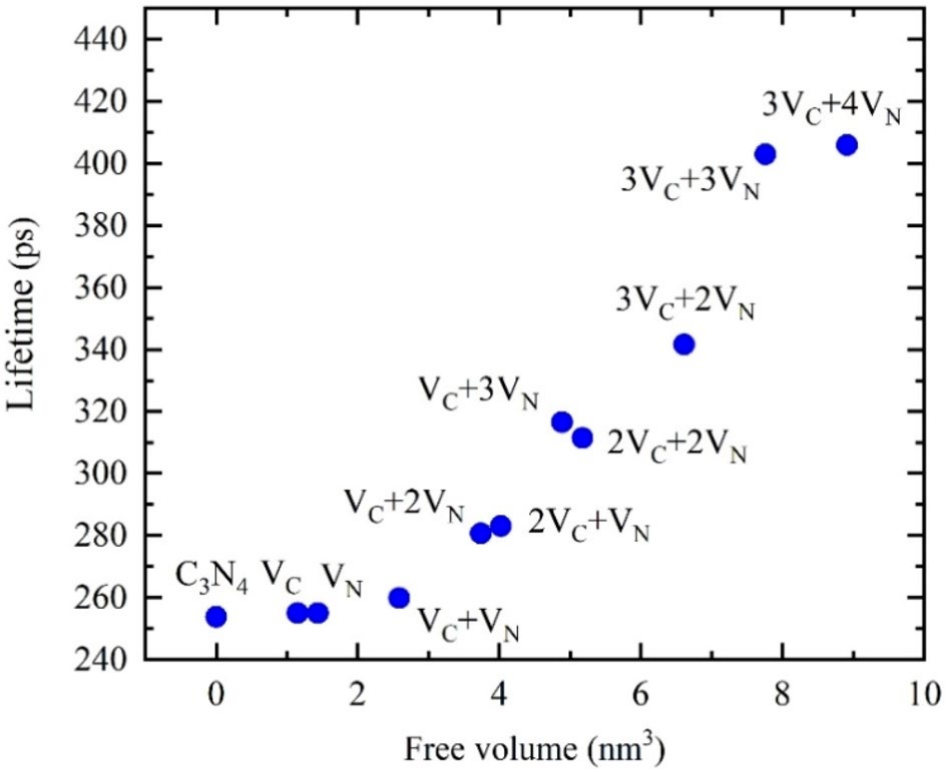
Calculated positron lifetimes for perfect C_3_N_4_ lattice (labelled C_3_N_4_) and for different types of point defects: carbon vacancies (V_C_), nitrogen vacancies (V_N_) and their complexes (nV_C_ + mV_N_).

The material morphology was studied by SEM. Figure 9 displays two micrographs of CN (left) and CH-Ar0 (right). Unlike the compact CN particles, the CN-Ar0 ones were composed of smaller fragments of various sizes and shapes with pores among them. The smaller fragments represent the smaller planes of C_3_N_4_ with more terminating > NH and –NH_2_ groups. The higher portion of these N–H species were observed by FTIR and the elemental analysis. The other CN-Ar materials resembled CN-Ar0 and are demonstrated in Figs. 12S and 13S.

### Mott-Schottky measurements

**Figure 14:**
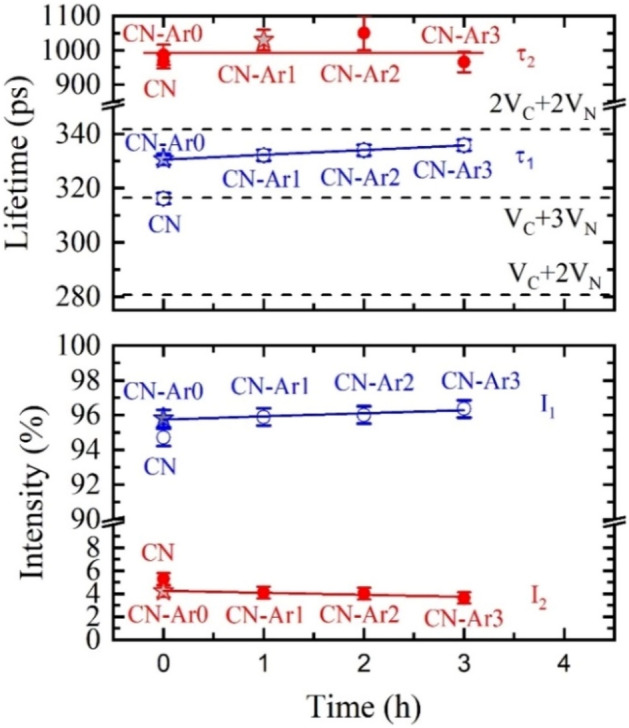
Dependence of lifetimes and intensities of individual components on annealing time in argon. Horizontal dashed lines show calculated lifetimes for different types of defects.

Flat band potentials of the CN and CN-Ar materials were measured against the Ag/AgCl reference electrode (see Fig. 14S) and recalculated to be against the normal hydrogen electrode (NHE) as E _vs. NHE_ = E _vs. Ag/AgCl_ + 0.191 V + 0.059 (7 – pH)^64^ yielding the values of − 0.97 V, − 0.83 V, − 0.83 V, − 0.91 V and − 0.88 V for CN, CN-Ar0, CN-Ar1, CN-Ar2, CN-Ar3 and CN-Ar4, respectively. Since the flat-band potential is about 0.2 V more positive than conductive band one^65^, the conduction band potentials were corrected at − 1.17 V, − 1.03 V, − 1.03 V, − 1.11 V and − 1.08 V, respectively. The corresponding valence band potentials (E_VB_) were calculated according to Eq. (5) and the results are summarized in Fig. 10.

5 $${\text{E}}_{{{\text{VB}}}} = {\text{ E}}_{{{\text{CB}}}} + {\text{ E}}_{{\text{g}}}$$

From this figure one can see that the conduction band as well as valence band potentials of the CN-Ar materials are similar. The E_VB_ values above 1.23 V indicate that these materials have the potential to be used in hydrogen generation by water splitting, see below.

### Photocatalytic activity

The photocatalytic activity of the CN-Ar materials was tested in terms of the generation of hydrogen by water splitting:

6 $${\text{2 H}}_{{2}} {\text{O }} \to {\text{ 2 H}}_{{2}} + {\text{ O}}_{{2}}$$

This reaction includes two redox chemical half reactions: Water oxidation half reaction

7 $${\text{2 H}}_{{2}} {\text{O }} + {\text{ 4 h}}^{ + } \to {\text{ O}}_{{2}} + {\text{ 4 H}}^{ + } \quad \left( {{\text{E}}^{0} = { 1}.{\text{23 eV}}} \right)$$

and proton reduction half reaction

8 $${\text{4 H}}^{ + } + {\text{ 4 e}}^{ - } \to {\text{ 2 H}}_{{2}} \quad \left( {{\text{E}}^{0} = \, 0{\text{ eV}}} \right)$$

The photocatalytic water splitting is an energy-intensive reaction. Therefore, the experiments were performed in the presence of electron donors (sacrificial reagents), such as methanol, to avoid back-reaction^66^.

Figure 11 shows the dependence of the hydrogen yields on the time (0–4 h) of irradiation (254 nm). A commercial TiO_2_ photocatalyst Evonik P25 was used for comparison. The CN material showed lower yields of hydrogen (547 µmol g^-1^) after 4 h of irradiation—by about half compared to TiO_2_ (1052 µmol g^-1^). On the other hand, all the CN-Ar materials generated more hydrogen than TiO_2_. Moreover, the highest yields of hydrogen were obtained in the presence of CN-Ar2 (1547 µmol g^-1^). Figure 12 shows the yields of produced hydrogen, methane and carbon monoxide (the CH_4_ and CO yields were multiplied by 10 for better recognition), after 4 h of irradiation. Methane and CO_2_ are typical intermediates of the photocatalytic decomposition of methanol^67^.

In general, the photocatalytic activity can be affected by several factors, such as phase composition, specific surface area, pore volumes, crystallite sizes, band gap energy, defects etc. The C/N ratio was found to play an important role. The elemental composition, which was determined by XPS, showed that the CN-Ar materials had a lower C/N ratio than CN that was prepared in air (Table 4). The CN one was of the highest C/N and its photoactivity was the lowest (547 μmol g^-1^ of H_2_), while CN-Ar2 and CN-Ar3 had the lowest C/N values and showed the highest photoactivity (1547 and 1482 μmol g^-1^ of H_2_, respectively). These findings indicate that the defects had a significant impact on the photocatalytic hydrogen generation.

Moreover, as shown in Fig. 2, CN exhibited the highest PL intensity, which means that the charge recombination was the highest and, therefore, less photogenerated charge carriers were available for the hydrogen generation. On the contrary, the lowest PL intensity was observed for the CN-Ar2 and CN-Ar3 ones, which had the highest photoactivity. This points out that the material defects confining the recombination of photoinduced electrons and holes have impact on reactions such as the hydrogen generation. The presence of defects was further analysed by PAS.

### Positron annihilation spectroscopy study

The positron lifetime spectrum of the CN material contains two components. The first component with the lifetime τ_1_ = 316 ps comes from positrons annihilating as particles, the second one with the lifetime τ_2_ = 990 ps comes from pick-off annihilation of ortho-positronium (o-Ps), i.e. a hydrogen-like bound state of positron and electron^68^. Note that a short para-positronium (p-Ps) component with the lifetime of 125 ps and intensity fixed to one-third of the intensity of the o-Ps component (corresponding to p-Ps to o-Ps branching ratio) was considered in the decomposition of PAS spectra.

The lifetime τ_1_ = 316 ps is significantly longer than the calculated bulk positron lifetime for C_3_N_4_, τ_B_ = 254 ps, i.e. the lifetime of free positrons delocalized in perfect (defect-free) C_3_N_4_ lattice. It indicates that positrons in the CN and CN-Ar materials were trapped in some open-volume defects. In order for us to identify these defects, the lifetimes of positrons trapped at various types of point defects in C_3_N_4_ lattice positron were calculated and are displayed in Fig. 13 as a function of open volume, representing a measure of defect ‘size’. From inspection of Fig. 13 one can conclude that the lifetime τ_1_ = 316 ps measured in CN is not only longer than the bulk positron lifetime but is also longer than the lifetime of positrons trapped in either carbon (V_C_) or nitrogen (V_N_) single vacancies. So, the CN material contains defects with a larger free volume than monovacancies. The lifetime τ_1_ = 316 ps measured in CN corresponds well to the calculated lifetime of positrons captured in V_C_ + 3V_N_ complexes. Considering nanocrystalline grain size of CN samples (see Table 2), which is more than an order of magnitude smaller than a typical positron diffusion length in solids^69^, a majority of positrons are annihilated at grain interfaces. Hence, V_C_ + 3V_N_ complexes are likely located at interfaces between crystallites.

The presence of a long component with the lifetime of τ_2_ = 990 ps originating from the o-Ps pick-up annihilation indicates that the material contains nanoscopic pores. Using the Tao-Eldrup model^70,71^ it is possible to estimate from the measured lifetime τ_2_ the mean size of nanoscopic pores of 3.2 ± 0.2 nm. Figure 14 shows the lifetimes and intensities of the individual components measured in the CN and CN-Ar materials and their dependence on the annealing time in argon. In CN-Ar0, the lifetime τ_1_ increased to ≈ 330 ps and with the increasing annealing time the lifetime increased further towards the value calculated for 2V_C_ + 2V_N_ complexes. Hence, the materials prepared in argon clearly contain larger defects and the size of these defects further increases during the heating in argon. It seems that V_C_ + 3V_N_ complexes (open volume of 4.89 nm^3^) tend to form larger complexes 2V_C_ + 2V_N_ (open volume of 5.17 nm^3^) containing two carbon vacancies. The lifetime τ_2_ is nearly constant during annealing in argon, without significant changes. The I_2_ intensity of the o-Ps component is smaller in CN-Ar0 than in CN and decreases slightly with heating. It follows from this that the CN-Ar materials contained a slightly lower concentration of nanoscopic pores.

**Figure 15:**
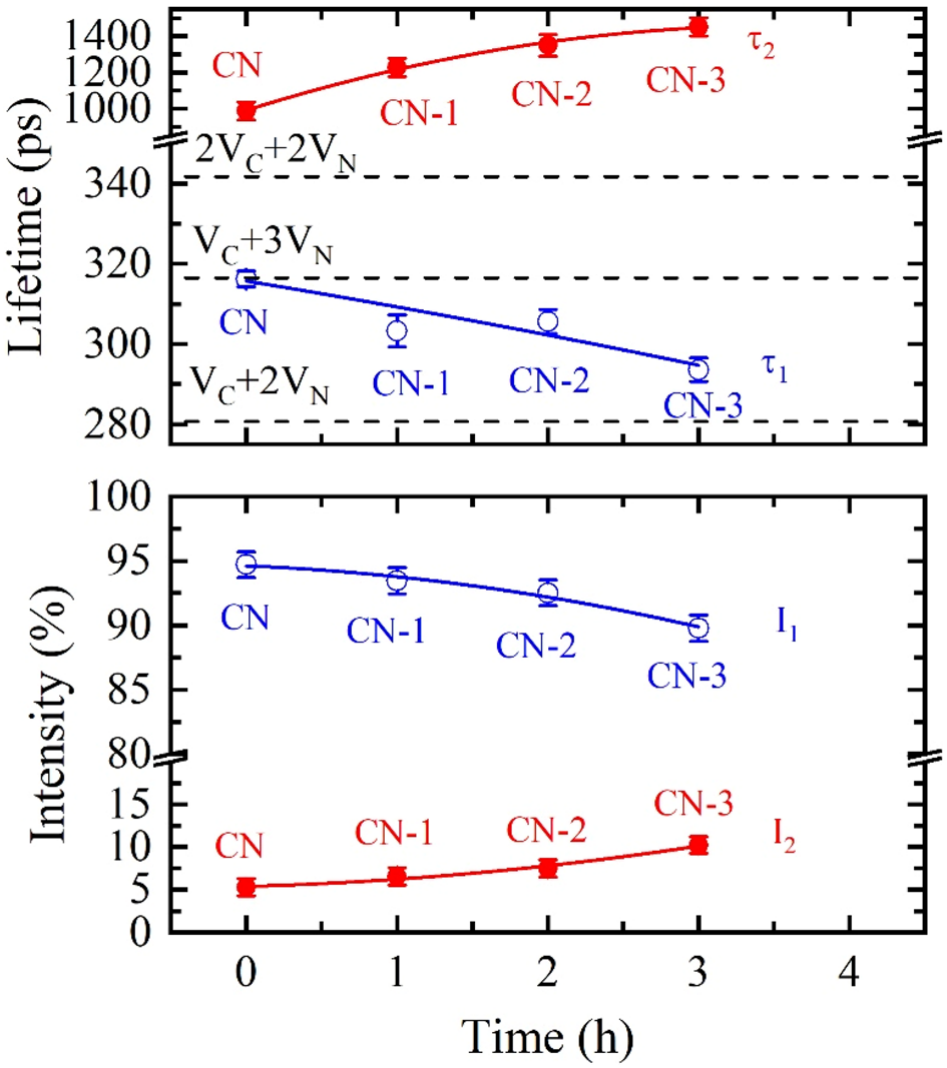
Dependence of lifetimes and intensities of individual components on annealing time in air^33^. Horizontal dashed lines show calculated lifetimes for different types of defects.

For comparison, the lifetimes and intensities of the CN materials synthetized and further heated in air^33^ are shown in Fig. 15 as well. Unlike the CN-Ar materials, these ones were exfoliated and one can see the opposite trends with respect to Fig. 14. The lifetime τ_1_ gradually decreases during exfoliation from 316 ps corresponding to V_C_ + 3V_N_ towards the value calculated for V_C_ + 2V_N_. At the same time, the intensity I_1_ decreases as well. The lifetime τ_2_ and the intensity I_2_ gradually increase during the exfoliation. It points to a gradual increase of size and concentration of nanoscopic pores. Using the Tao-Eldrup model^70,71^ one can estimate that the mean size of nanoscopic pores increased from 3.2 ± 0.2 nm to 4.6 ± 0.2 nm during the exfoliation for 3 h. This finding is in agreement with the loss of nitrogen during the repeated heating for 1–3 h observed by XPS. Unlike the C_3_N_4_ synthesis and further heating in argon, the synthesis and heating in air are accompanied by reactions with oxygen and, hence, the vacancy complexes are formed by different mechanisms, which are still unclear. The defect number reduction in CN and CN-1 to CN-3 observed by the PL spectrometry is likely a part of this process.

The PAS analysis demonstrated that one cannot talk about either nitrogen or carbon single vacancies but about their complexes. The longer heating in argon resulted in the higher vacancy volumes (Fig. 13). This is in consistency with the C/N values as well as the increasing photocatalytic activity of the CN-Ar materials and it also agrees with other authors who referred to the enhanced photocatalytic generation of hydrogen due to the presence of N vacancies in C_3_N_4_^30,32,34–37,54,63^.

### Effect of inert gas on synthesis of C_3_N_4_

The synthesis of C_3_N_4_ was based on the polycondensation of melamine forming heptazine and then melon units, which were mutually connected into the planes. This process is necessarily associated with the release of ammonia, which must be removed in order to shift the reaction equilibria toward the resulting C_3_N_4_ in line with Le Chatelier’s principle. During the synthesis, ammonia diffused out through the already formed porous C_3_N_4_ and, at the same time, surrounding argon diffused in the opposite direction, see Fig. 15S.

In general, this situation, when two gases of different molecular masses are diffusing in the opposite directions through a porous media, can be described by Graham´s law of gas diffusion as follows:

9 $$\frac{{v_{NH3} }}{{v_{Ar} }} = \sqrt {\frac{{M_{Ar} }}{{M_{NH3} }}}$$

where *v*_*NH3*_ and *v*_*Ar*_ are the rates of diffusion of NH_3_ and Ar, respectively; *M*_*NH3*_ and *M*_*A*r_ are the molar masses of NH_3_ and Ar, respectively. Substituting for the molar masses M_Ar_ = 40 g mol^−1^, M_NH3_ = 17 g mol^−1^ and M_N2_ = 28 g mol^−1^ we can get from this relationship that the rate of NH_3_ is 1.53 times higher than the rate of Ar and the rate of NH_3_ is 1.28 times higher than that of N_2_.

The diffusion rates of gases can be expressed by means of their number of moles diffusing through a porous medium during the same time. The total number of diffusing NH_3_ (*n*_*NH3*_) and Ar (*n*_*Ar*_) moles is n = n_NH3_ + n_Ar_, which we can substitute in Eq. (9) as

10 $$\frac{{v_{NH3} }}{{v_{Ar} }} = \frac{{n_{NH3} }}{{n_{Ar} }} = \frac{{n_{NH3} }}{{n - n_{NH3} }} = \sqrt {\frac{{M_{Ar} }}{{M_{NH3} }}}$$

Then, we can calculate the molar fraction of NH_3_ defined as

11 $$x_{NH3} = \left( {1 + \sqrt {\frac{{M_{NH3} }}{{M_{Ar} }}} } \right)^{ - 1}$$

Substituting for the molar masses of Ar and NH_3_ we can get x_NH3_ = 0.61. Using the same equation for N_2_ and NH_3_ we can get x_NH3_ = 0.56. Thus, we can see that the number of moles of released NH_3_ is higher in the argon than in nitrogen atmosphere.

Considering the reaction equilibria, it can be supposed that more ammonia released means more complete C_3_N_4_ and less incomplete C_3_N_4_ labelled as C_x_N_y_H_z_ in Fig. 15S. In our previous work, the content of oxygen in C_3_N_4_ synthesised under nitrogen was 7.52 wt. %^33^ in contrast to 2.28 wt. % determined in this work (Table 5). The incomplete C_x_N_y_H_z_ is supposed to be more reactive with oxygen than C_3_N_4_ due to various structural defects including vacancies. The higher content of the defects in C_x_N_y_H_z_ was indicated by the higher C/N values of 0.686–0.700 (mol/mol)^33^, see Table 5 for comparison.

Equation (11) is an approximate calculation, which helps us to understand the beginning phase of the C_3_N_4_ synthesis in an inert atmosphere. In a real process, it is also possible to consider the reaction rate of synthesis, which was changing with the increasing temperature, and complex geometry and a temperature field of resulting C_3_N_4_ in a crucible.

## Conclusion

Graphitic carbon nitride was synthesised at 550 °C for 4 h in the argon atmosphere and then heated for 1–3 h in argon again. The UV–Vis reflectance spectra revealed the two band gap energies of 2.04 eV (608 nm) and 2.47 eV (502 nm). The photoluminescence study indicated the formation of defects during the heating, which explains the band gap of 2.04 eV. Although the defects were formed, the regular C_3_N_4_ structure was preserved as demonstrated by XRD and FTIR. The CN-Ar materials were not exfoliated by their heating; their SSAs were 15–18 m^2^g^−1^. Macropores were formed in the CN-Ar materials as calculated by the BJH model and observed by SEM.

The bulk elemental analysis confirmed the presence of nitrogen defects likely due to the loss of the N_2C_ atoms. It was also observed that all the materials were photoactive because they were able to generate photoelectric current around the wavelength of 380 nm. Using the Mott-Schottky plots similar conduction band potentials of CN and CN-Ar materials were observed. The positions of valence band potentials indicated their capability of the photocatalytic water splitting, which was tested in terms of hydrogen generation in a water–methanol mixture. The best CN-Ar3 photocatalyst showed 3 times higher performance (1547 μmol g^-1^) compared to the CN photocatalyst (547 μmol g^-1^) and 1.5 higher compared to reference TiO_2_ (1052 μmol g^-1^).

The PAS analysis showed the formation of the complexes of carbon and nitrogen vacancies. Big 2V_C_ + 2V_N_ and Vc + 3V_N_ complexes were formed in argon in contrast to Vc + 3V_N_ ones formed in air. The presence of the 2V_C_ + 2V_N_ complexes is assumed to affect the photocatalytic activity of C_3_N_4_. This positive effect resulted in higher hydrogen generation performance using CN-Ar materials in comparison to the CN one. Finally, the effect of an inert gas on the synthesis of graphitic carbon nitride from nitrogen-rich precursors was demonstrated based on Graham´s law of gas diffusion.

It was shown that not only the presence of vacancies, but also their size is the important factor of the C_3_N_4_ photocatalytic activity for hydrogen generation. The research focused on vacancy complexes in C_3_N_4_ will be performed in the future. The investigation based on theoretical calculations and material characterizations could lead to deeper understanding of the physico-chemical properties of C_3_N_4_ structures for their prospective applications.

## Supplementary Information

Supplementary Information.

## Data Availability

The datasets used and/or analysed during the current study available from the corresponding author on reasonable request.

## References

[1] Liu AY, Cohen ML (1989). Prediction of new low compressibility solids. Science.

[2] Wang X (2009). A metal-free polymeric photocatalyst for hydrogen production from water under visible light. Nat. Mater..

[3] Zhang H, Zuo X, Tang H, Li G, Zhou Z (2015). Origin of photoactivity in graphitic carbon nitride and strategies for enhancement of photocatalytic efficiency: Insights from first-principles computations. Phys. Chem. Chem. Phys..

[4] Wang Y, Wang X, Antonietti M (2012). Polymeric graphitic carbon nitride as a heterogeneous organocatalyst: From photochemistry to multipurpose catalysis to sustainable chemistry. Angew. Chem. Int. Ed. Engl..

[5] Cao S, Low J, Yu J, Jaroniec M (2015). Polymeric photocatalysts based on graphitic carbon nitride. Adv. Mater..

[6] Yang Y (2020). Recent advances in application of graphitic carbon nitride-based catalysts for degrading organic contaminants in water through advanced oxidation processes beyond photocatalysis: A critical review. Water Res..

[7] Safaei J (2018). Graphitic carbon nitride (g-C_3_N_4_) electrodes for energy conversion and storage: A review on photoelectrochemical water splitting, solar cells and supercapacitors. J. Mater. Chem. A.

[8] Dong Y (2016). Graphitic carbon nitride materials: Sensing, imaging and therapy. Small.

[9] Wang A, Wang C, Fu L, Wong-Ng W, Lan Y (2017). Recent advances of graphitic carbon nitride-based structures and applications in catalyst, sensing, imaging, and LEDs. Nano-Micro Lett..

[10] Wang L, Wang C, Hu X, Xue H, Pang H (2016). Metal/graphitic carbon nitride composites: Synthesis, structures, and applications. Chem. Asian J..

[11] Zhou Z, Zhang Y, Shen Y, Liu S, Zhang Y (2018). Molecular engineering of polymeric carbon nitride: Advancing applications from photocatalysis to biosensing and more. Chem. Soc. Rev..

[12] Svoboda L (2018). Graphitic carbon nitride nanosheets as highly efficient photocatalysts for phenol degradation under high-power visible LED irradiation. Mater. Res. Bull..

[13] Wang Y, Liu L, Ma T, Zhang Y, Huang H (2021). 2D graphitic carbon nitride for energy conversion and storage. Adv. Funct. Mater..

[14] Papailias I (2018). Chemical vs thermal exfoliation of g-C_3_N_4_ for NO_x_ removal under visible light irradiation. Appl. Catal. B.

[15] Jiang L (2017). Doping of graphitic carbon nitride for photocatalysis: A reveiw. Appl. Catal. B.

[16] Starukh H, Praus P (2020). Doping of graphitic carbon nitride with non-metal elements and its application for photocatalysis. Catalysts.

[17] Hasija V (2019). Recent advances in noble metal free doped graphitic carbon nitride based nanohybrids for photocatalysis of organic contaminants in water: A review. Appl. Mater. Today.

[18] Fu J, Yu J, Jiang C, Cheng B (2018). g-C_3_N_4_-based heterostructured photocatalysts. Adv. Energy Mater..

[19] Miller TS (2017). Carbon nitrides: Synthesis and characterization of a new class of functional materials. Phys. Chem. Chem. Phys..

[20] Ong W-J, Tan L-L, Ng YH, Yong S-T, Chai S-P (2016). Graphitic carbon nitride (g-C_3_N_4_)-based photocatalysts for artificial photosynthesis and environmental remediation: Are we a step closer to achieving sustainability?. Chem. Rev..

[21] Kessler FK (2017). Functional carbon nitride materials—design strategies for electrochemical devices. Nat. Rev. Mater..

[22] Kong L, Wang J, Ma F, Sun M, Quan J (2019). Graphitic carbon nitride nanostructures: Catalysis. Appl. Mater. Today.

[23] Inagaki M, Tsumura T, Kinumoto T, Toyoda M (2019). Graphitic carbon nitrides (g-C_3_N_4_) with comparative discussion to carbon materials. Carbon.

[24] Li Y, Li X, Zhang H, Fan J, Xiang Q (2020). Design and application of active sites in g-C3N4-based photocatalysts. J. Mater. Sci. Technol..

[25] Barrio J, Volokh M, Shalom M (2020). Polymeric carbon nitrides and related metal-free materials for energy and environmental applications. J. Mater. Chem. A.

[26] Zhu B, Zhang L, Cheng B, Yu J (2018). First-principle calculation study of tri-s-triazine-based g-C_3_N_4_: A review. Appl. Catal. B.

[27] Wang Y (2019). Catalysis with two-dimensional materials confining single atoms: Concept, design, and applications. Chem. Rev..

[28] Lv C (2018). Defect engineering metal-free polymeric carbon nitride electrocatalyst for effective nitrogen fixation under ambient conditions. Angew. Chem. Int. Ed..

[29] Niu P, Yin L-C, Yang Y-Q, Liu G, Cheng H-M (2014). Increasing the visible light absorption of graphitic carbon nitride (melon) photocatalysts by homogeneous self-modification with nitrogen vacancies. Adv. Mater..

[30] Tu W (2017). Investigating the role of tunable nitrogen vacancies in graphitic carbon nitride nanosheets for efficient visible-light-driven H_2_ evolution and CO_2_ reduction. ACS Sustain. Chem. Eng..

[31] Liao J (2020). Nitrogen defect structure and NO^+^ intermediate promoted photocatalytic NO removal on H_2_ treated g-C3N4. Chem. Eng. J..

[32] Liang L (2019). Synthesis and photo-catalytic activity of porous g-C_3_N_4_: Promotion effect of nitrogen vacancy in H_2_ evolution and pollutant degradation reactions. Int. J. Hydrogen Energy.

[33] Praus P (2020). The presence and effect of oxygen in graphitic carbon nitride synthetized in air and nitrogen atmosphere. Appl. Surf. Sci..

[34] Liang L, Shi L, Wang F (2019). Fabrication of large surface area nitrogen vacancy modified graphitic carbon nitride with improved visible-light photocatalytic performance. Diam. Relat. Mater..

[35] Barrio J (2018). Unprecedented centimeter-long carbon nitride needles: Synthesis. Charact. Appl..

[36] Jiménez-Calvo P, Marchal C, Cottineau T, Caps V, Keller V (2019). Influence of the gas atmosphere during the synthesis of g-C_3_N_4_ for enhanced photocatalytic H_2_ production from water on Au/g-C_3_N_4_ composites. J. Mater. Chem. A.

[37] Huang S (2021). Synthesis of carbon nitride in moist environments: A defect engineering strategy toward superior photocatalytic hydrogen evolution reaction. J. Energy Chem..

[38] Jiang L (2021). Defect engineering in polymeric carbon nitride photocatalyst: Synthesis, properties and characterizations. Adv. Coll. Interface Sci..

[39] Yu X (2021). Point-defect engineering: leveraging imperfections in graphitic carbon nitride (g-C3N4) photocatalysts toward artificial photosynthesis. Small.

[40] Kumar A (2021). C-, N-Vacancy defect engineered polymeric carbon nitride towards photocatalysis: Viewpoints and challenges. J. Mater. Chem. A.

[41] Yang J (2022). Defective polymeric carbon nitride: Fabrications, photocatalytic applications and perspectives. Chem. Eng. J..

[42] Tauc J, Grigorovici R, Vancu A (1966). Optical properties and electronic structure of amorphous germanium. Phys. Status Solidi (b).

[43] Xu Y, Gao S-P (2012). Band gap of C_3_N_4_ in the GW approximation. Int. J. Hydrogen Energy.

[44] Bečvář F, Čížek J, Procházka I, Janotová J (2005). The asset of ultra-fast digitizers for positron-lifetime spectroscopy. Nucl. Instrum. Methods Phys. Res. Sect. A.

[45] Čížek J (2020). PLRF code for decomposition of positron lifetime spectra. Acta Phys. Pol. A.

[46] Puska MJ, Nieminen RM (1994). Theory of positrons in solids and on solid surfaces. Rev. Mod. Phys..

[47] Puska MJ, Nieminen RM (1983). Defect spectroscopy with positrons: a general calculational method. J. Phys. F Met. Phys..

[48] Barbiellini B, Puska MJ, Torsti T, Nieminen RM (1995). Gradient correction for positron states in solids. Phys. Rev. B.

[49] Barbiellini B (1996). Calculation of positron states and annihilation in solids: A density-gradient-correction scheme. Phys. Rev. B.

[50] Zuo H-W, Lu C-H, Ren Y-R, Li Y, Zhang Y-F, Chen W-K (2016). Pt_4_ clusters supported on monolayer graphitic carbon nitride sheets for oxygen adsorption: A first-principles study. Acta Phys. Chim. Sin..

[51] Zhang X, Zhao M, Wang A, Wang X, Du A (2013). Spin-polarization and ferromagnetism of graphitic carbon nitride materials. J. Mater. Chem. C.

[52] Dias EM, Christoforidis KC, Francàs L, Petit C (2018). Tuning thermally treated graphitic carbon nitride for H_2_ evolution and CO_2_ photoreduction: The effects of material properties and mid-gap states. ACS Appl. Energy Mater..

[53] Zhang Y (2013). Synthesis and luminescence mechanism of multicolor-emitting g-C_3_N_4_ nanopowders by low temperature thermal condensation of melamine. Sci. Rep..

[54] Wu P, Wang J, Zhao J, Guo L, Osterloh FE (2014). Structure defects in g-C_3_N_4_ limit visible light driven hydrogen evolution and photovoltage. J. Mater. Chem. A.

[55] Liang X (2019). Graphitic carbon nitride with carbon vacancies for photocatalytic degradation of bisphenol A. ACS Appl. Nano Mater..

[56] Wan L, Egerton RF (1996). Preparation and characterization of carbon nitride thin films. Thin Solid Films.

[57] Mendes RG (2021). Tailoring the stoichiometry of C_3_N_4_ nanosheets under electron beam irradiation. Phys. Chem. Chem. Phys..

[58] Sanagi MM (2009). Comparison of signal-to-noise, blank determination, and linear regression methods for the estimation of detection and quantification limits for volatile organic compounds by gas chromatography. J. AOAC Int..

[59] Thomas A (2008). Graphitic carbon nitride materials: Variation of structure and morphology and their use as metal-free catalysts. J. Mater. Chem..

[60] Komatsu T (2001). The first synthesis and characterization of cyameluric high polymers. Macromol. Chem. Phys..

[61] Li Y (2020). C_3_N_4_ with engineered three coordinated (N_3C_) nitrogen vacancy boosts the production of ^1^O_2_ for Efficient and stable NO photo-oxidation. Chem. Eng. J..

[62] Wu J (2019). Nitrogen vacancies modified graphitic carbon nitride: Scalable and one-step fabrication with efficient visible-light-driven hydrogen evolution. Chem. Eng. J..

[63] Niu P, Liu G, Cheng H-M (2012). Nitrogen vacancy-promoted photocatalytic activity of graphitic carbon nitride. J. Phys. Chem. C.

[64] Giannakopoulou T (2017). Tailoring the energy band gap and edges’ potentials of g-C3N4/TiO2 composite photocatalysts for NOx removal. Chem. Eng. J..

[65] Shen R, Liu W, Ren D, Xie J, Li X (2019). Co1.4Ni0.6P cocatalysts modified metallic carbon black/g-C3N4 nanosheet Schottky heterojunctions for active and durable photocatalytic H2 production. Appl. Surf. Sci..

[66] Kočí K (2018). Photocatalytic decomposition of methanol over La/TiO2 materials. Environ. Sci. Pollut. Res..

[67] Miller TL, Wolin MJ (1983). Oxidation of hydrogen and reduction of methanol to methane is the sole energy source for a methanogen isolated from human feces. J. Bacteriol..

[68] Mogensen OE (1995). Positron Annihilation in Chemistry.

[69] Hugenschmidt C (2016). Positrons in surface physics. Surf. Sci. Rep..

[70] Tao SJ (1972). Positronium annihilation in molecular substances. J. Chem. Phys..

[71] Eldrup M, Lightbody D, Sherwood JN (1981). The temperature dependence of positron lifetimes in solid pivalic acid. Chem. Phys..

