# Supplementary material for: Synthesis of vacant graphitic carbon nitride in argon atmosphere and its utilization for photocatalytic hydrogen generation
Source: Sci Rep. 2022 Aug 10;12:13622. doi: 10.1038/s41598-022-17940-3 (PMC9365785; doi:10.1038/s41598-022-17940-3)
Supplement: Supplementary file 1 — Supplementary Information. [file 41598_2022_17940_MOESM1_ESM.docx]

**Supplementary materials**

**Synthesis of vacant graphitic carbon nitride in argon atmosphere and its utilization for photocatalytic hydrogen generation**

Petr Praus^1,2,*^, Lenka Řeháčková^1^, Jakub Čížek^3^, Aneta Smýkalová^1,2^, Martin Koštejn^4^, Jiří Pavlovský^1^, Miroslava Filip Edelmanová^2^, Kamila Kočí^2^

^1^Department of Chemistry and Physico-Chemical processes, VŠB-Technical University of Ostrava, 17. listopadu 15, 708 00 Ostrava-Poruba, Czech Republic

^2^Institute of Environmental Technology, CEET, VŠB-Technical University of Ostrava, 17. listopadu 15, 708 00 Ostrava-Poruba, Czech Republic

^3^Department of Low-Temperature Physics, Faculty of Mathematics and Physics, Charles University, V Holesovickach 2, Prague 8, Czech Republic

^4^Institute of Chemical Process Fundamentals, Czech Academy of Science, Rozvojová 1,

165 02 Prague, Czech Republic

*Corresponding author: petr.praus@vsb.cz

Table 1S. Band gap energies CN materials synthesised in air

| Material | E_g_ (eV) | SSA (m^2^ g^-1^) |
| --- | --- | --- |
| CN | 2.69 | 12 |
| CN-1 | 2.72 | 58 |
| CN-2 | 2.75 | 90 |
| CN-3 | 2.77 | 142 |

Table 2S. Basic XRD characteristics of CN materials synthesised in air

| Material | 2 Theta (deg) | FWHM (deg) | L(002) (nm) | d(002) (nm) |
| --- | --- | --- | --- | --- |
| CN | 31.91 | 1.30 | 7.1 | 0.325 |
| CN-1 | 32.11 | 1.34 | 6.9 | 0.323 |
| CN-2 | 32.18 | 1.35 | 6.8 | 0.323 |
| CN-3 | 32.17 | 1.38 | 6.7 | 0.323 |

Table 3S. Ratios of FTIR absorbances of CN synthesised in air

| Material | A_3163_/A_1242_ | A_3163_/A_1637_ | A_1242_/A_1637_ |
| --- | --- | --- | --- |
| CN | 0.402 | 0.450 | 1.12 |
| CN-1 | 0.341 | 0.431 | 1.26 |
| CN-2 | 0.381 | 0.471 | 1.24 |
| CN-3 | 0.386 | 0.469 | 1.21 |

Table 4S. Ratios of FTIR absorbances of CN and CN-Ar materials

| Material | A_3435_/A_1242_ | A_34350_/A_1637_ |
| --- | --- | --- |
| CN | 0.232 | 0.260 |
| CN-Ar0 | 0.308 | 0.341 |
| CN-Ar1 | 0.367 | 0.401 |
| CN-Ar2 | 0.271 | 0.301 |
| CN-Ar3 | 0.376 | 0.413 |

Table 5S. Bulk elemental analysis of CN materials synthesised in air

| Material | C (wt %) | N (wt %) | H (wt %) | O (wt %) | C/N (mol/mol) |
| --- | --- | --- | --- | --- | --- |
| CN | 34.57 | 61.57 | 1.58 | 2.28 | 0.655 |
| CN-1 | 33.90 | 60.50 | 2.25 | 3.35 | 0.654 |
| CN-2 | 33.60 | 60.00 | 2.27 | 4.13 | 0.653 |
| CN-3 | 33.60 | 59.90 | 2.24 | 4.26 | 0.654 |


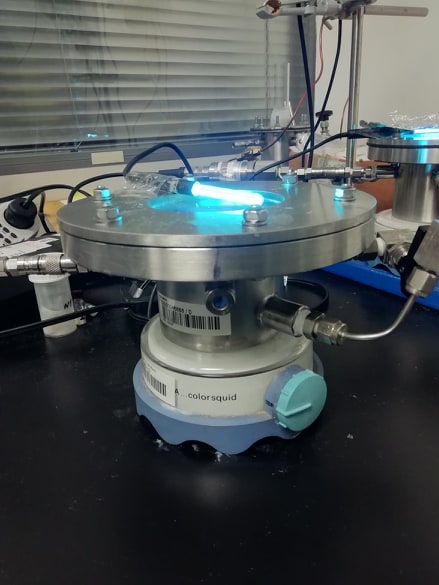


**a)**

Figure 1S Batch reactor used for photocatalytic hydrogen generation.


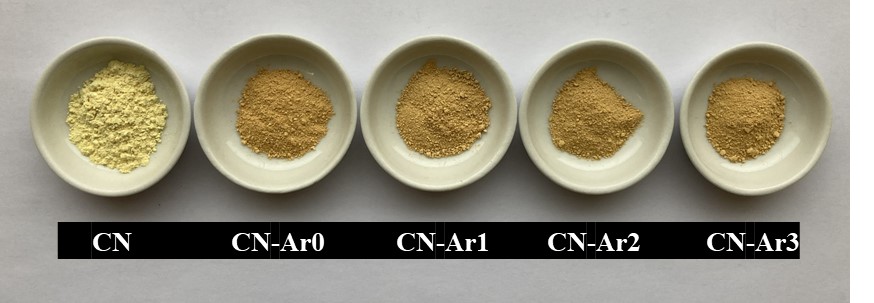


Figure 2S. Colours of CN and CN-Ar materials.


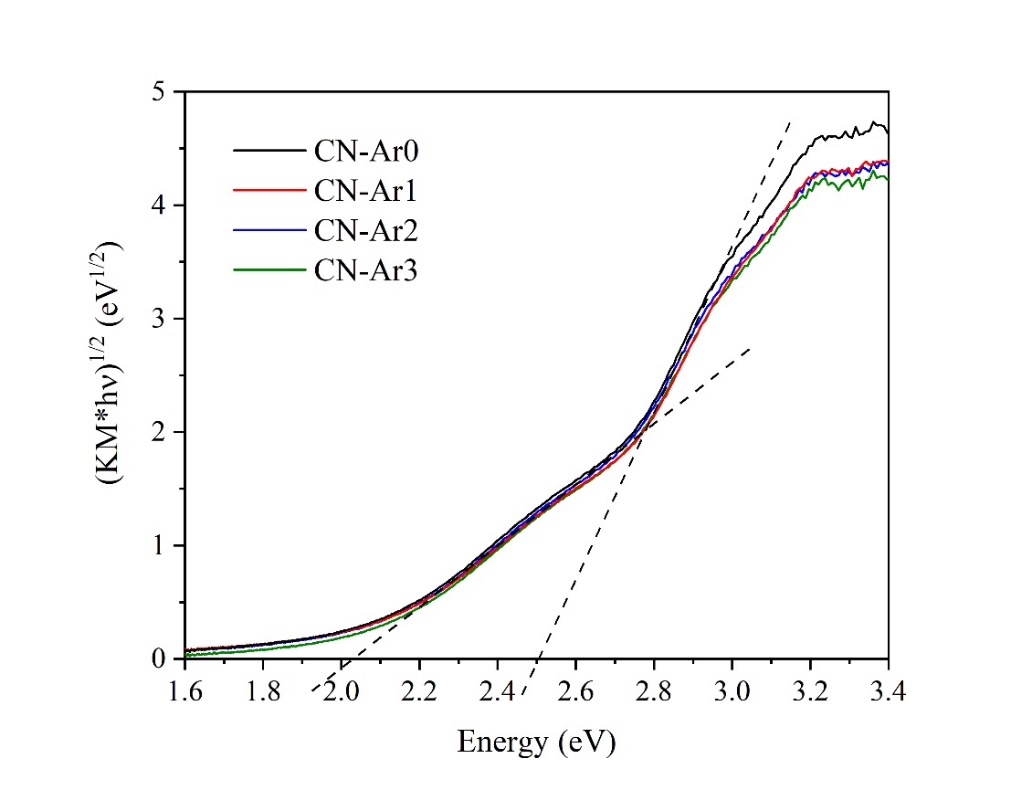


Figure 3S. Tauc plots of CN-Ar materials.


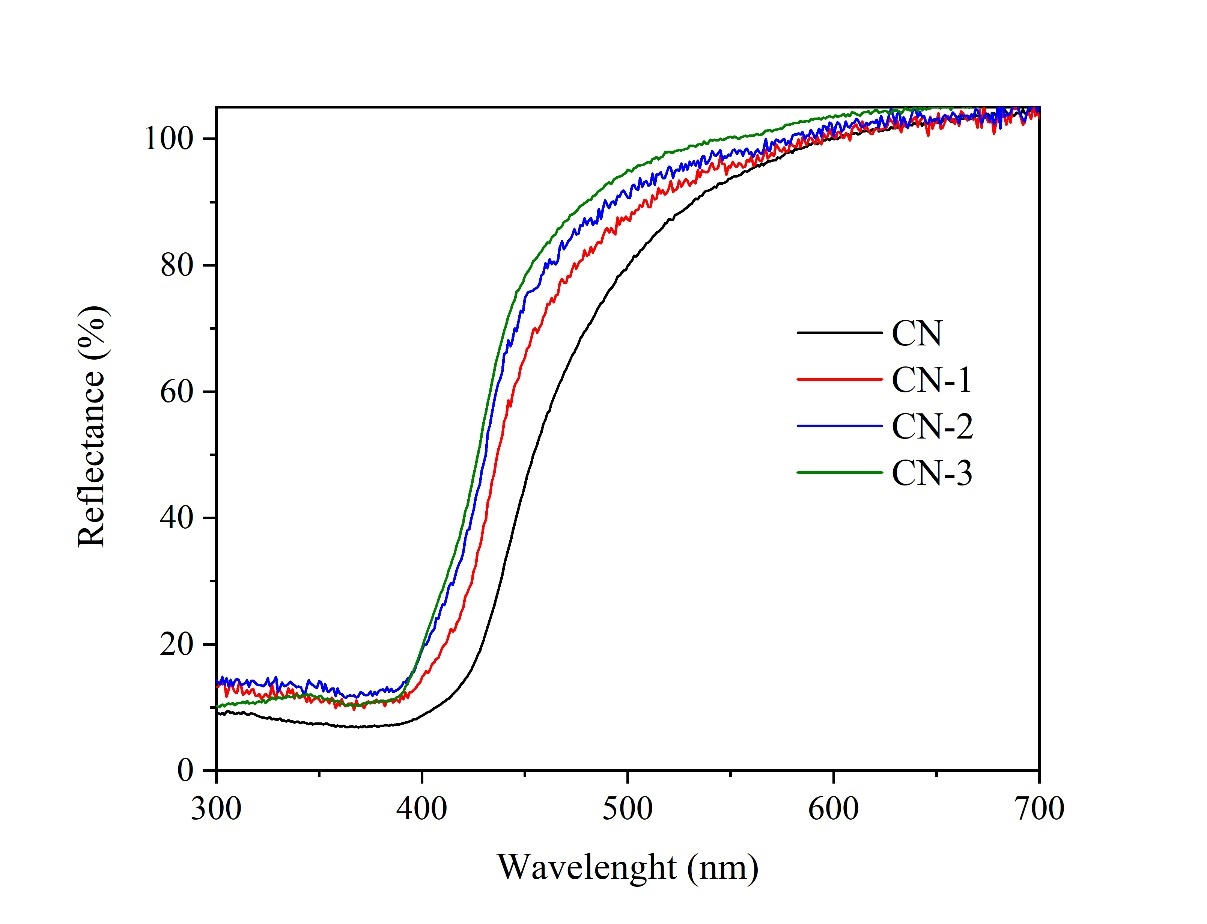


Figure 4S UV-Vis reflectance spectra of CN materials synthesised in air.


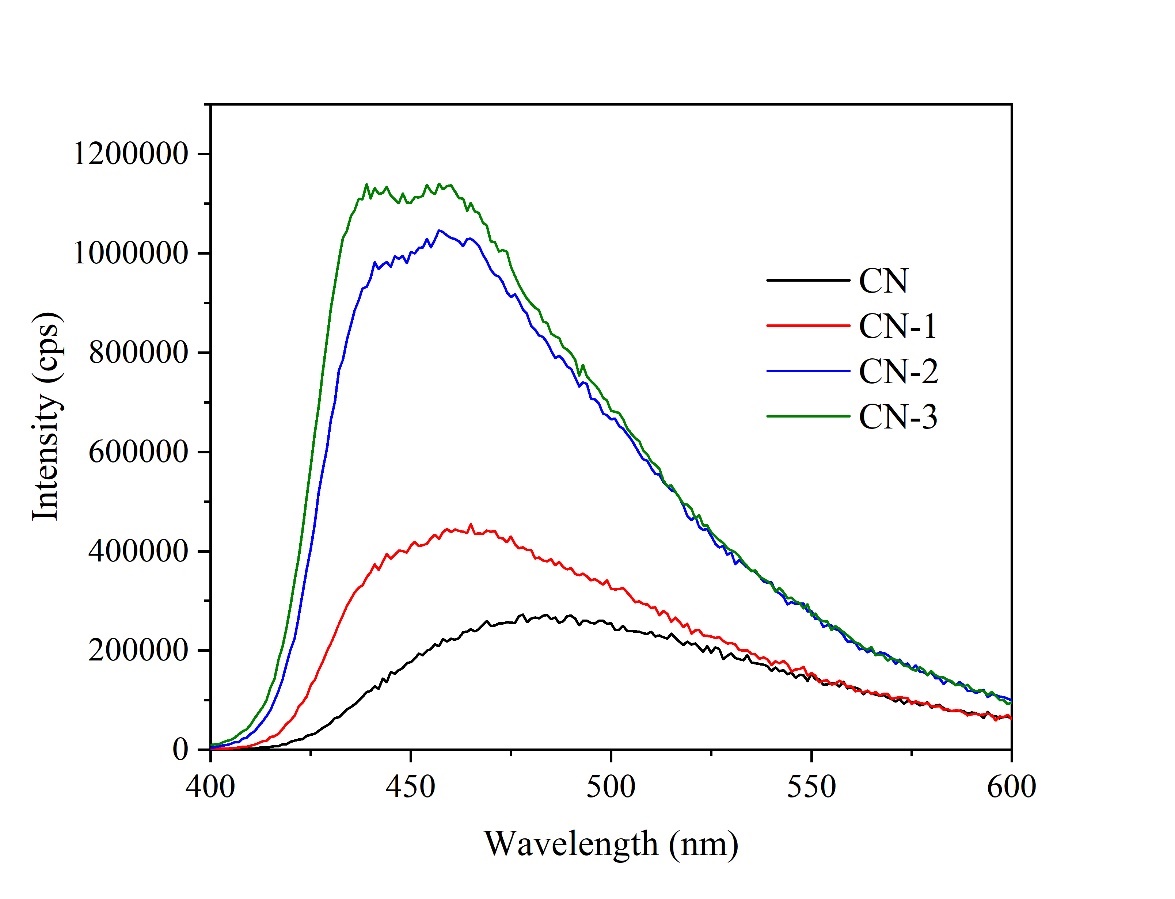


Figure 5S. PL spectra of CN materials synthesised in air.


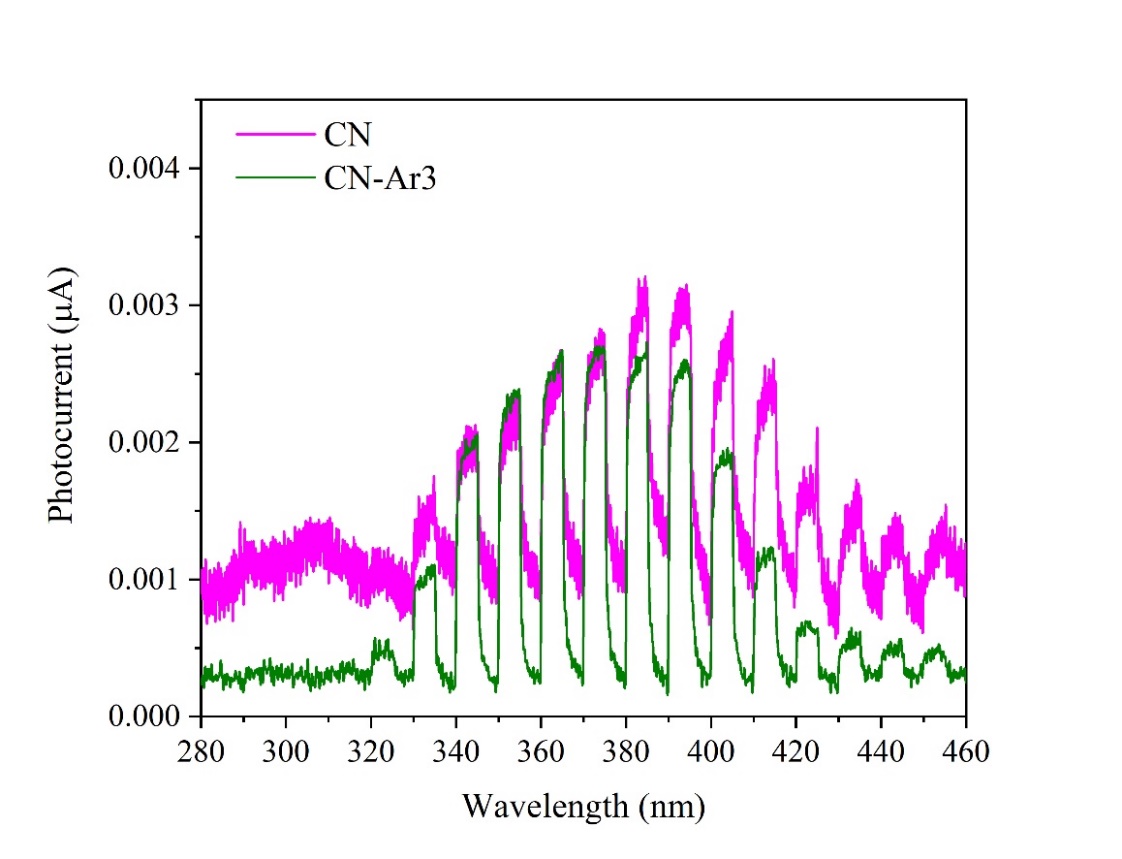


Figure 6S. Photocurrents of CN and CN-Ar3 materials recorded at 1V vs. Ag/AgCl in deoxygenated 0.1 mol L^-1^ KNO3.


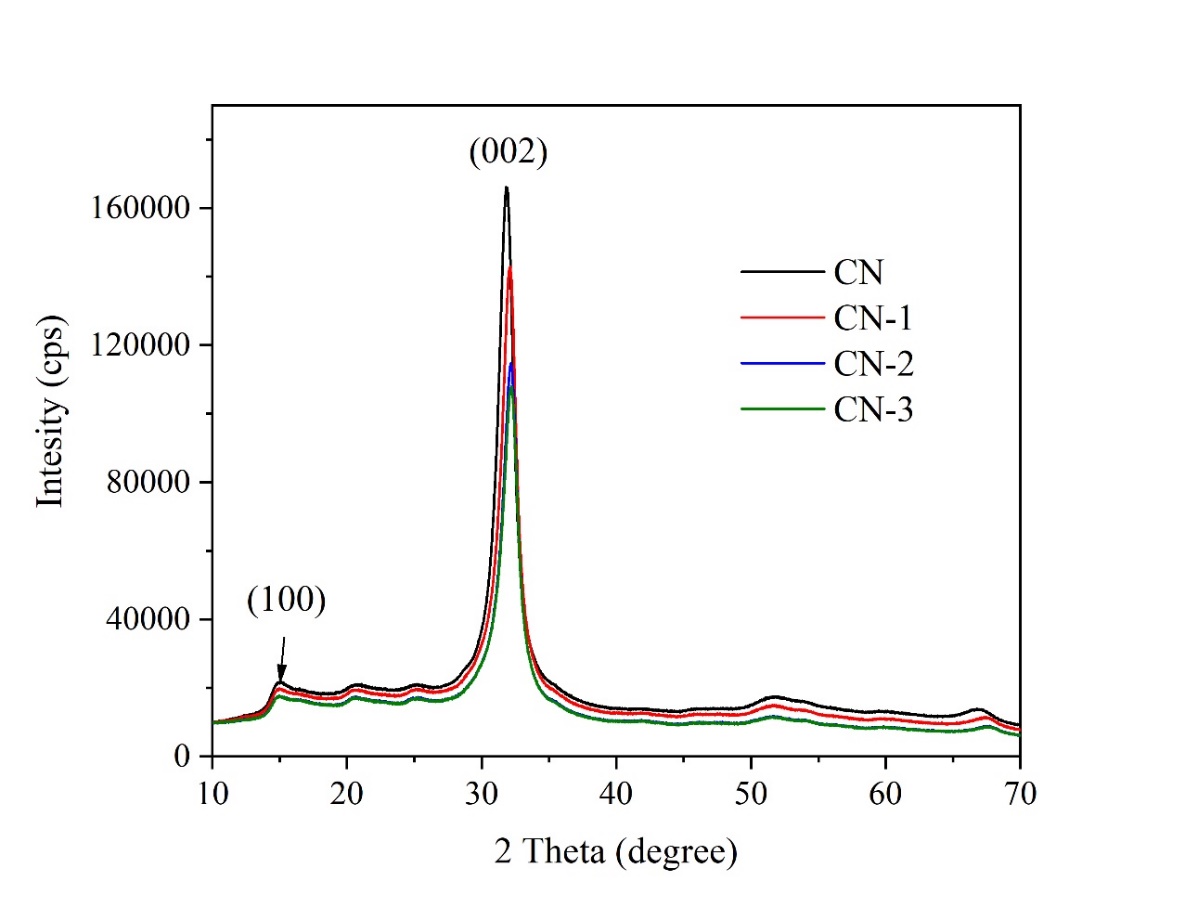


Figure 7S. XRD patterns of CN materials synthesised in air.


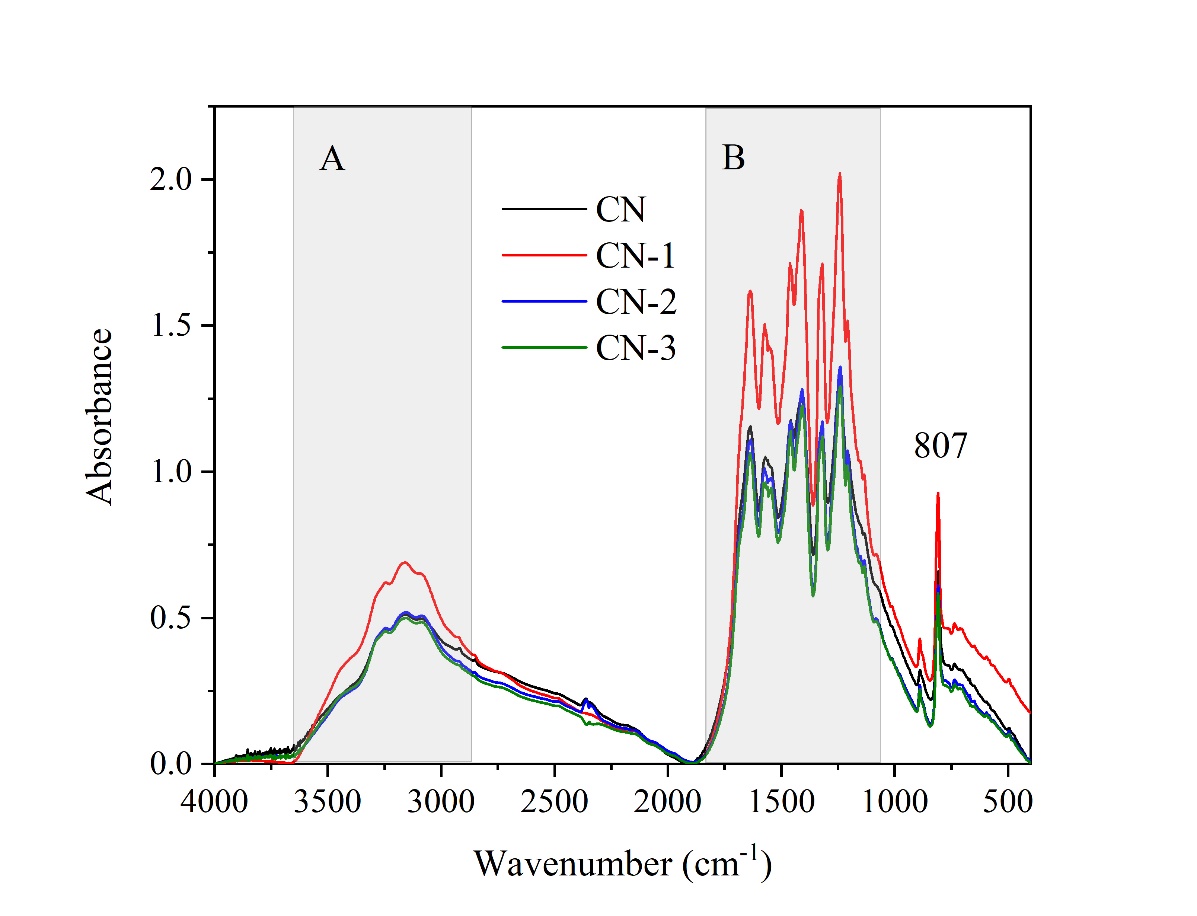


Figure 8S. FTIR spectra of CN materials synthesised in air.


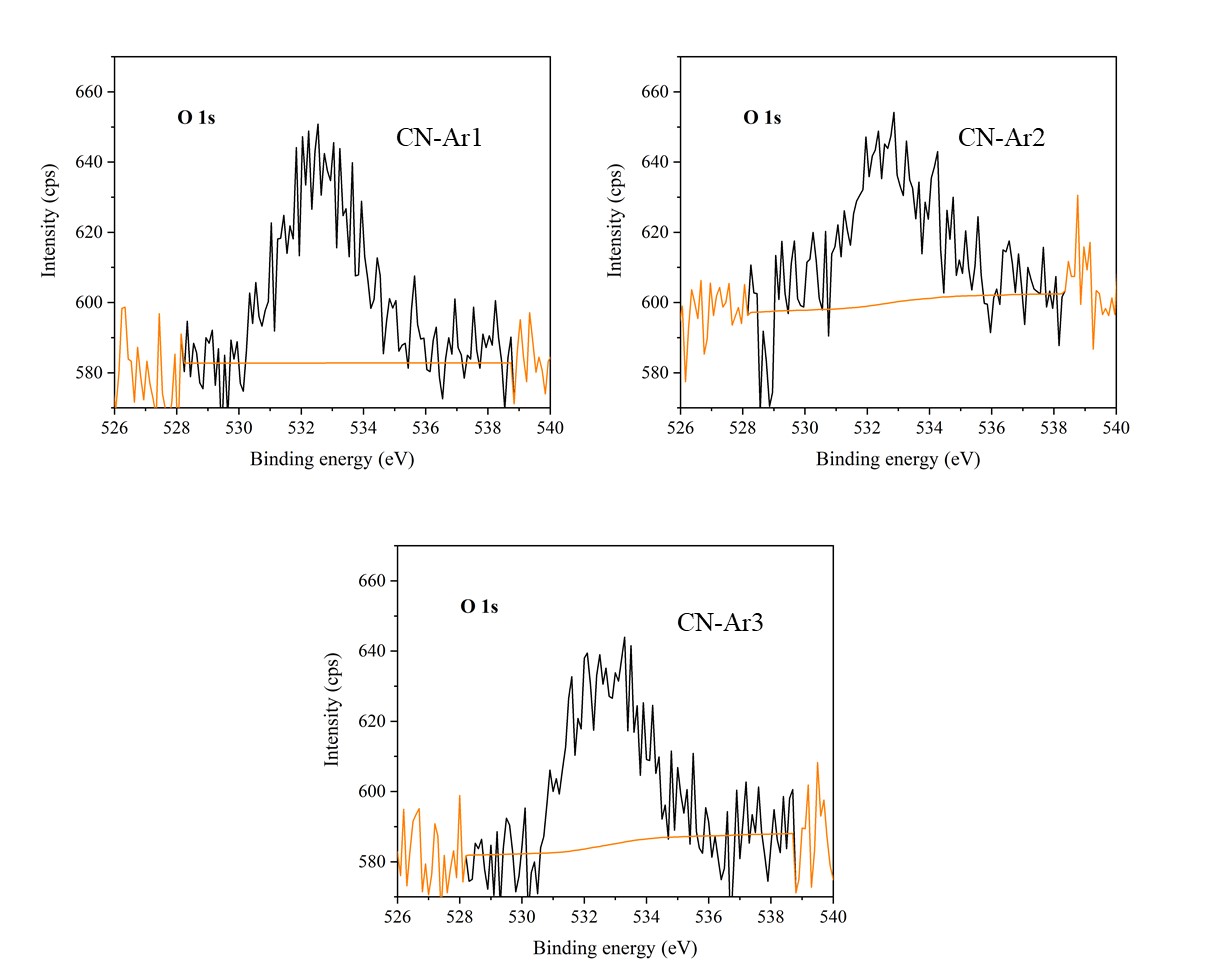


Figure 9S. XPS O 1s spectra of CN-Ar1, CN-Ar2, and CN-Ar3.


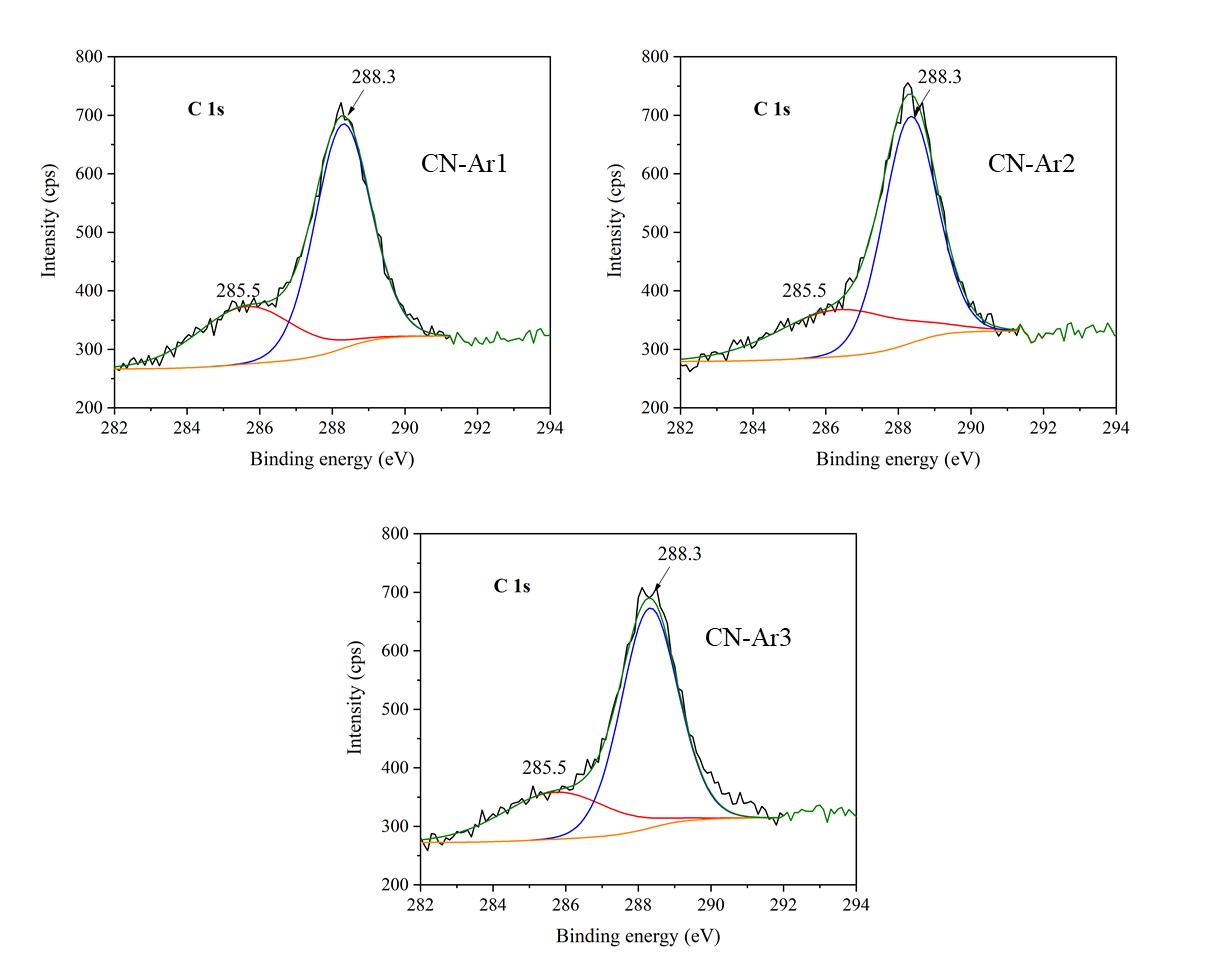


Figure 10S. XPS C 1s spectra of CN-Ar1, CN-Ar2, and CN-Ar3.


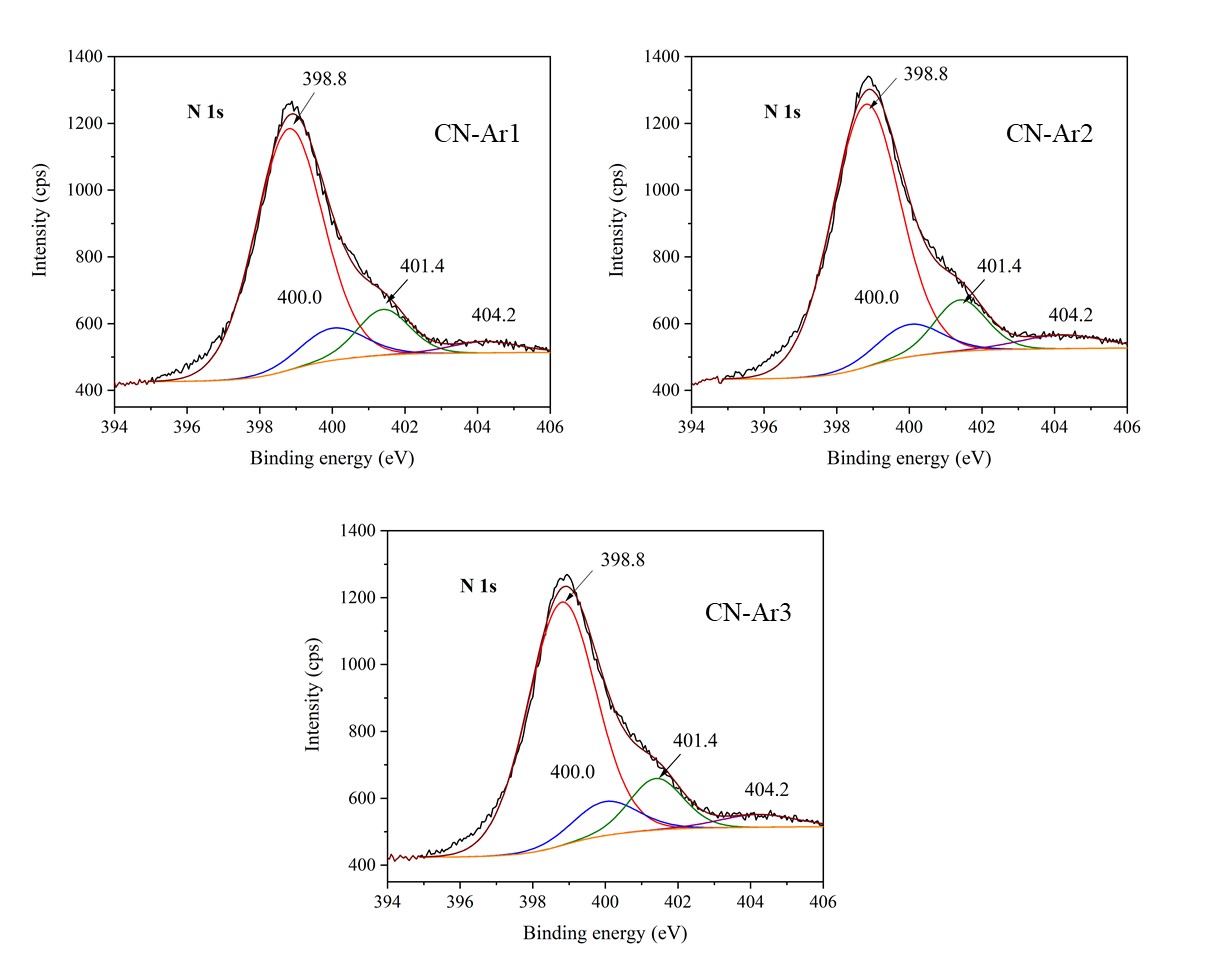


Figure 11S. XPS N 1s spectra of CN-Ar1, CN-Ar2, and CN-Ar3.


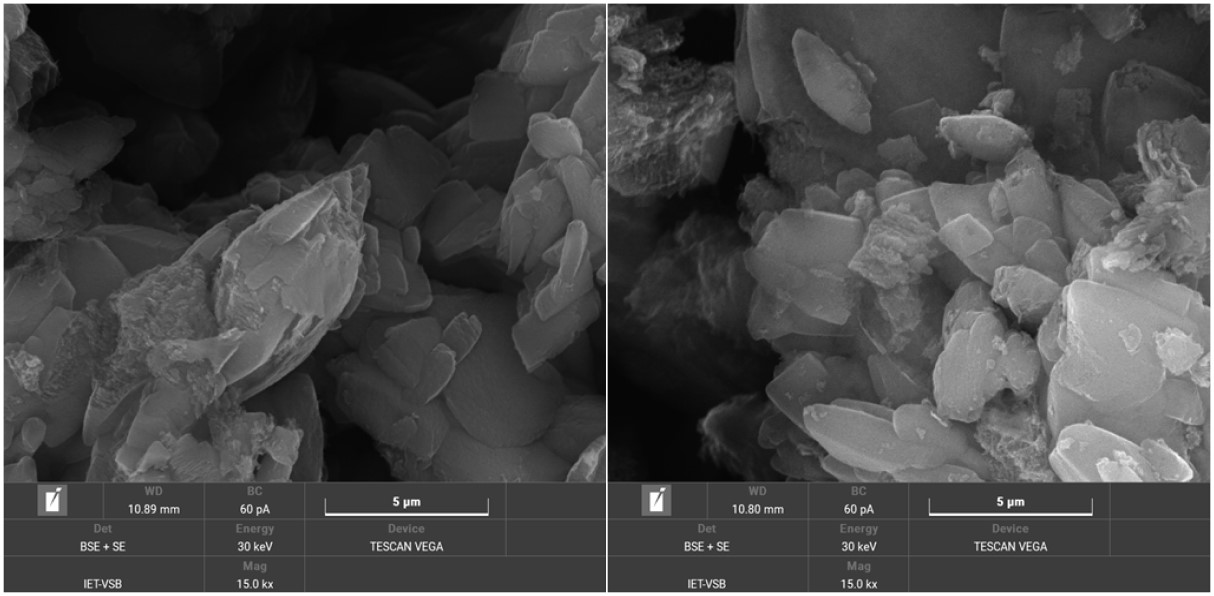


Figure 12S. SME (BSE+SE) micrographs of CN-Ar1 (left) and CN-Ar2 (right).


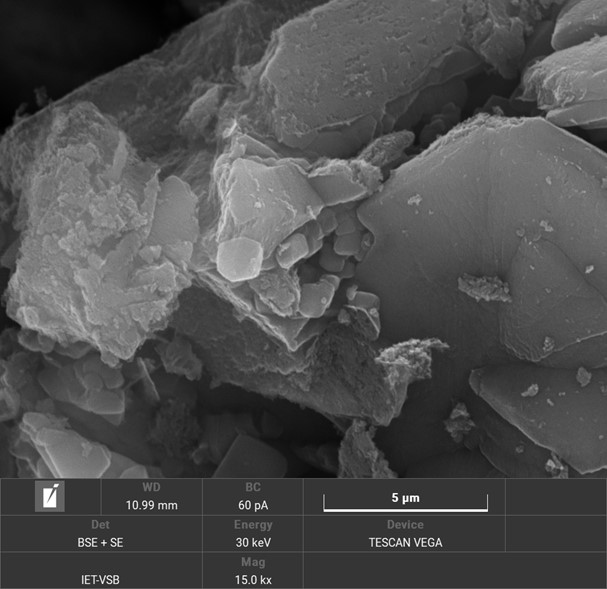


Figure 13S. SME (BSE+SE) micrograph of CN-Ar3.

Figure 14S Mott-Schottky plots of CN and CN-Ar materials measured at 300 Hz.


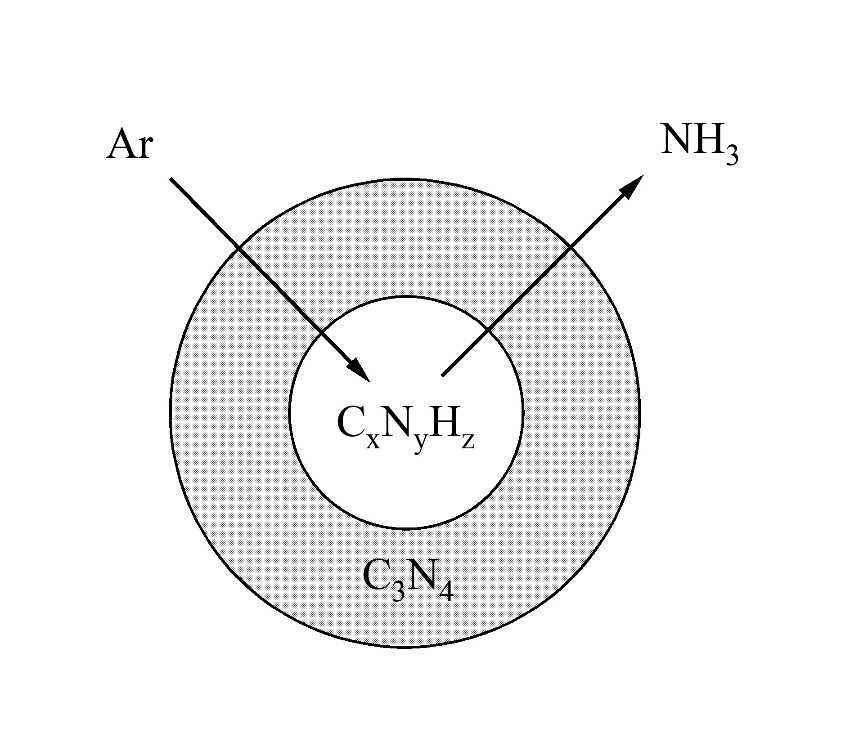


Figure 15S. Diffusion of NH_3_ and Ar during synthesis of C_3_N_4_ from C_x_N_y_H_z_.
